# DNA alteration‐based classification of uveal melanoma gives better prognostic stratification than immune infiltration, which has a neutral effect in high‐risk group

**DOI:** 10.1002/cam4.2122

**Published:** 2019-04-25

**Authors:** Deepti Narasimhaiah, Catherine Legrand, Diane Damotte, Romain Remark, Marco Munda, Patrick De Potter, Pierre G. Coulie, Miikka Vikkula, Catherine Godfraind

**Affiliations:** ^1^ Human Molecular Genetics de Duve Institute, Université catholique de Louvain Brussels Belgium; ^2^ Institute of Statistics, Biostatistics and Actuarial Sciences Université catholique de Louvain Louvain‐la‐Neuve Belgium; ^3^ Team “Cancer, Immune control, and Escape”, Centre de Recherche des Cordeliers INSERM U1138 Paris France; ^4^ Department of Ophthalmology Université catholique de Louvain Brussels Belgium; ^5^ de Duve Institute Université catholique de Louvain Brussels Belgium; ^6^ Department of Pathology CHU Gabriel Montpied Clermont‐Ferrand France

## Abstract

**Background:**

In uveal melanomas, immune infiltration is a marker of poor prognosis. This work intended to decipher the biological characteristics of intra‐tumor immune population, compare it to other established biomarkers and to patients' outcome.

**Methods:**

Primary, untreated, and mainly large uveal melanomas with retinal detachment were analyzed using: transcriptomic profiling (n = 15), RT‐qPCR (n = 36), immunohistochemistry (n = 89), Multiplex Ligation‐dependent Probe Amplification (MLPA) for copy number alterations (CNA) analysis (n = 89), array‐CGH (n = 17), and survival statistics (n = 86).

**Results:**

Gene expression analysis divided uveal melanomas into two groups, according to the IFNγ/STAT1‐IRF1 pathway activation. Tumors with IFNγ‐signature had poorer prognosis and showed increased infiltration of CD8^+^ T lymphocytes and macrophages. Cox multivariate analyses of immune cell infiltration with MLPA data delineated better prognostic value for three prognostic groups (three‐tier stratification) than two (two‐tier stratification). CNA‐based model comprising monosomy 3, 8q amplification, and *LZTS1*and *NBL1* deletions emerged as the best predictor for disease‐free survival. It outperformed immune cell infiltration in receiver operating characteristic curves. The model that combined CNA and immune infiltration defined risk‐groups according to the number of DNA alterations. Immune cell infiltration was increased in the high‐risk group (73.7%), where it did not correlate with patient survival, while it was associated with poorer outcome in the intermediate risk‐group.

**Conclusions:**

High degree of immune cell infiltration occurs in a subset of uveal melanomas, is interferon‐gamma‐related, and associated with poor survival. It allows for two‐tier stratification, which is prognostically less efficient than a three‐tier one. The best prognostic stratification is by CNA model with three risk‐groups where immune cell infiltration impacts only some subgroups.

## 1 INTRODUCTION

The omics era has ushered in a better understanding of the molecular underpinnings of tumors that have resulted in identification of molecular subgroups within each cancer type. This has led to development of treatments targeting the underlying genetic alterations with better patient stratification and improved survival rates in many solid tumors. Unfortunately, uveal melanomas (UM) have lagged behind in this aspect, with a 5‐year survival rate that has not improved during the past three decades^1^ and liver metastases remain the major cause of mortality.

Few prognostic markers have been proposed for UM. Among them, monosomy 3 (M3) has for long been the most accepted one even though it leaves some tumors in between classes.^2^, ^3^ This two‐tier classification was improved by adding a second biomarker, 8q amplification that allowed a three‐tier stratification.^4^ Nevertheless, there is a need for better biomarkers, preferably ones applicable for aspiration biopsies. There are also lacunae with regard to the role of immune infiltrate as a biomarker, and its prognostic relevance when compared to genetic and clinical parameters. Here, we addressed these shortcomings by combining copy number alterations with transcriptomic profiling, expression of selected immune genes, and immunohistochemistry for immune cell infiltration.

Various modeling strategies are used for biomarker identification in oncology, with Cox proportional hazards being the most common one.^5^ The relative performances of the constructed models are assessed using methods such as time‐dependent receiver operating characteristic (ROC) curves^6^ and a secondary validation. The latter assesses whether the model works satisfactorily for a different patient cohort than the one used to develop the model. For routine clinical application, the model can be converted into a user‐friendly graphical version called nomogram.^7^

In this study, we have applied these methods to generate risk‐stratification models on a series of 91 primary, untreated, large choroidal and ciliochoroidal melanomas, most of them with retinal detachment.

## 2 MATERIALS AND METHODS

### 2.1 Tumor series

The series included 91 primary, previously untreated uveal (choroidal and ciliochoroidal) melanomas from patients who underwent enucleation at the Ophthalmology Department of the Cliniques universitaires Saint‐Luc, Brussels, Belgium from 1997 to 2010 (Table S1, Supplementary methods). Snap‐frozen (n = 15), RNA later preserved (n = 21), and formalin‐fixed paraffin‐embedded (FFPE, n = 89) tumor materials were available. The study was carried out according to the ethics committee guidelines of the Cliniques universitaires Saint‐Luc, Brussels, Belgium.

### 2.2 RNA extraction

Total RNA was extracted from 15 snap‐frozen and 21 RNA later samples, as described in Supplementary methods.

### 2.3 Transcriptomic analysis

RNA from the 15 snap‐frozen samples was processed as described previously.^8^ Gene expression profiles generated from Human Genome U133 Plus 2.0 arrays (Affymetrix, Santa Clara, CA) were processed using Microarray suite 5.0 gene expression software. Statistical analysis was performed using TIGR MultiExperiment Viewer platform (MeV4.8.1, http://www.tm4.org/mev/) and InnateDB (http://www.innatedb.com/) (Supplementary methods).

### 2.4 Immune gene list

A catalog of immunologically relevant genes was compiled from https://immport.niaid.nih.gov, http://amigo.geneontology.org and http://wiki.geneontology.org/index.php/Immunology (Supplementary methods).

### 2.5 Immunohistochemistry

FFPE sections from 89 uveal melanomas were immunolabeled with antibodies to CD3, CD4, CD8, CD163, and HLA‐DRA using BenchMark XT (Ventana Medical Systems, Tuscon, AZ) and GBP1 (n = 39) (Supplementary methods, Table M1).

### 2.6 Immune score calculation

Immune score was calculated by applying a semi‐quantitative scoring to HLA‐DRA and CD3‐stained slides. Five intra‐tumoral regions: edges (both), base, center, and apex were assessed. Depending on the extent of positive staining, a score from 0 to 2 was assigned to each of the five regions for HLA‐DRA and CD3 separately. The immune score was the sum of individual scores for HLA‐DRA and CD3.

### 2.7 Quantitative real‐time reverse transcription polymerase chain reaction

The real‐time reverse transcription polymerase chain reaction (RT‐qPCR) was performed using SYBR Green I master mix (catalog #04707516001) in a LightCycler 480 (both from Roche Diagnostics, Basel, Switzerland, Supplementary methods). The target genes tested were: CXCL9, GBP1, RARRES3, STAT1‐beta‐transcript, and PSMB9, and a reference gene, CSNK2B (Table M2).

### 2.8 Multiplex ligation‐dependent probe amplification and comparative genomic hybridization

Copy number status for chromosomes 1p, 3, 6 and 8 was analyzed (n = 89) using the SALSA MLPA kit P027‐C1 (MRC Holland, Amsterdam, Netherlands). Data were obtained with GeneMarker v2.2 software (Softgenetics, State College, PA) after population normalization, and tumor‐to‐reference ratio calculation. Ratios of ≤0.88 and ≥1.24 were used for loss and gain, respectively. These cut‐offs were derived from tumors (n = 17) assessed by both multiplex ligation‐dependent probe amplification (MLPA) and comparative genomic hybridization (CGH) (OncoScan™ FFPE Express 2.0 Services, Affymetrix, Supplementary methods).

### 2.9 Statistical analyses

**Figure 1:**
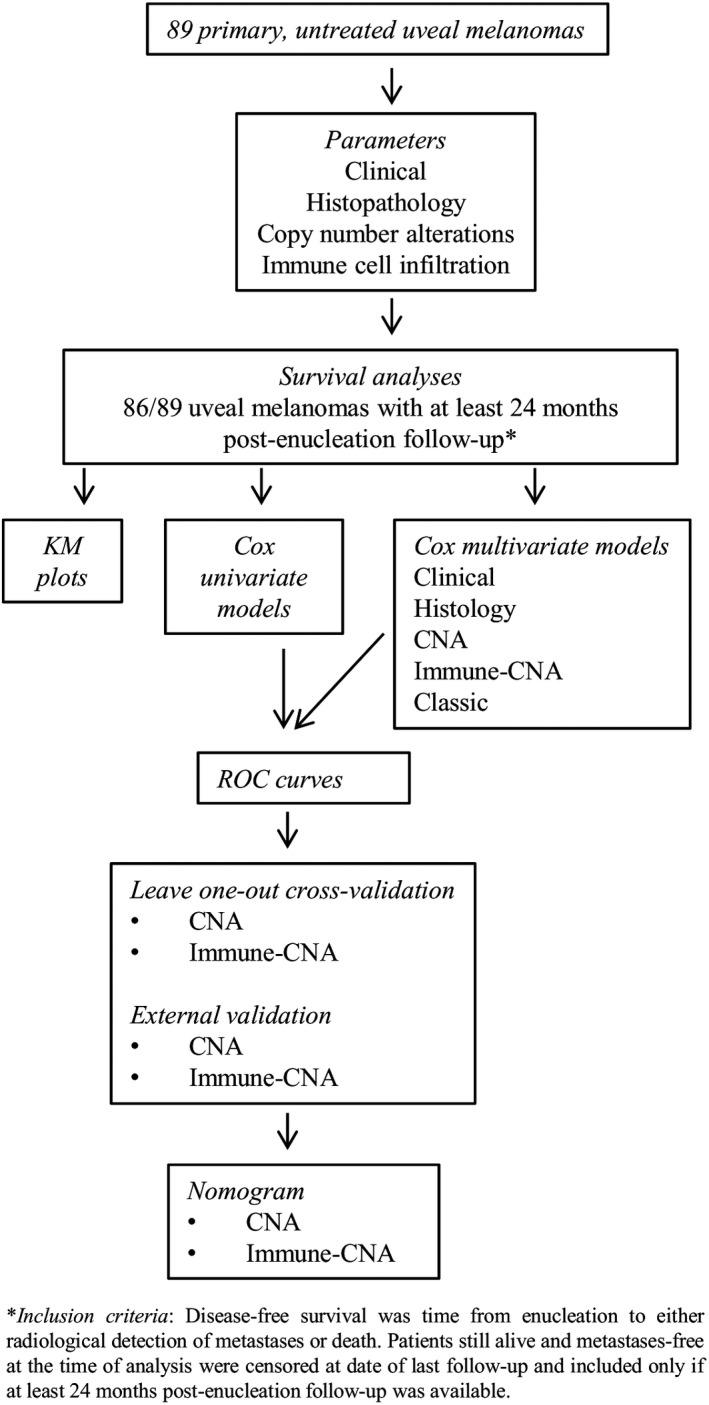
Scheme of statistical analyses

The scheme of statistical analyses is shown in Figure 1. Survival analyses (n = 86) were based on disease‐free survival (DFS). Kaplan‐Meier (KM) plots, Cox univariate and multivariate proportional hazards regression models were constructed. The models were assessed by ROC curves, leave‐one‐out cross‐validation, and nomograms were created. The tumors were divided into three or four risk‐groups using prognostic indices, the details of which are provided in Supplementary methods/Table M3. In addition, external validation on the The Cancer Genome Atlas (TCGA) project data.^9^ (Supplementary methods, Table M4) was performed. All statistical tests were two‐sided; *P* ≤ 0.05 was considered significant, and no multiplicity adjustment was made considering the exploratory nature of these analyses. The analyses were performed in IBM SPSS Statistics v20, v21 (IBM, Armonk, NY) and R software v2.15 (http://CRAN.R-project.org) using risksetROC and rms packages.

## 3 RESULTS

### 3.1 Immune signature based on gene expression

#### 3.1.1 Microarray analysis

**Figure 2:**
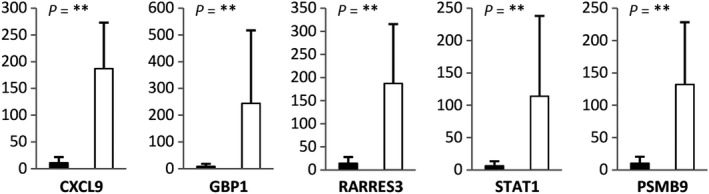
Validation of differentially expressed genes identified by microarray analyses in immune infiltrate‐high (n = 5, ⎕) and immune infiltrate‐low (n = 10, ▀) uveal melanomas (*P*‐value: ** <0.01)

Transcriptomic profiling was carried out on a set of 15 randomly selected primary, untreated uveal melanomas (UM). Hierarchical clustering (HCL), an unsupervised analysis separated the 15 tumors in two subgroups of five and ten, with dendrogram node values of 96 and 59, respectively (Figure S1a). Thirty‐four genes (44 probesets) identified by high‐stringency *T* test were responsible for this segregation (Figure S1a, Table S2), and were all relatively overexpressed in the subgroup of five tumors. As these genes were related to immune response in DAVID,^10^ the subgroups were referred to as “immune gene‐high” and “immune gene‐low”. This division was validated by RT‐qPCR for genes CXCL9, GBP1, RARRES3, PSMB9, and STAT1 with important fold differences (11.9‐34.3) and absolute values (693.5‐1953) of expression (*P* = 0.002, Mann‐Whitney test, Figure 2).

#### 3.1.2 Immune gene analysis

To expand the list of significant genes, immune response genes were compiled from various databases and submitted to HCL. The UM was separated into the same two subgroups, by 122 significant genes (156 probesets) identified by low‐stringency *T* test. Among them, 111 were relatively overexpressed and 11 underexpressed in the immune gene‐high subgroup (Figure S1b, Table S2). Twenty‐eight overexpressed genes were common with microarray analysis.

#### 3.1.3 Pathways and gene ontology terms associated with uveal melanoma subgroups

The 111 overexpressed genes were significantly associated with pathways corresponding to immune system, antigen processing and presentation, graft‐versus‐host disease and interferon‐gamma signaling (Table S3). Their related Gene Ontology (GO) terms included: immune response, innate immune response, antigen processing, and presentation of peptide antigen via MHC class I, and interferon‐gamma‐mediated signaling (Table S4).

### 3.2 Immune cell infiltration assessed by immunohistochemistry

#### 3.2.1 Definition of an IHC immune score

**Figure 3:**
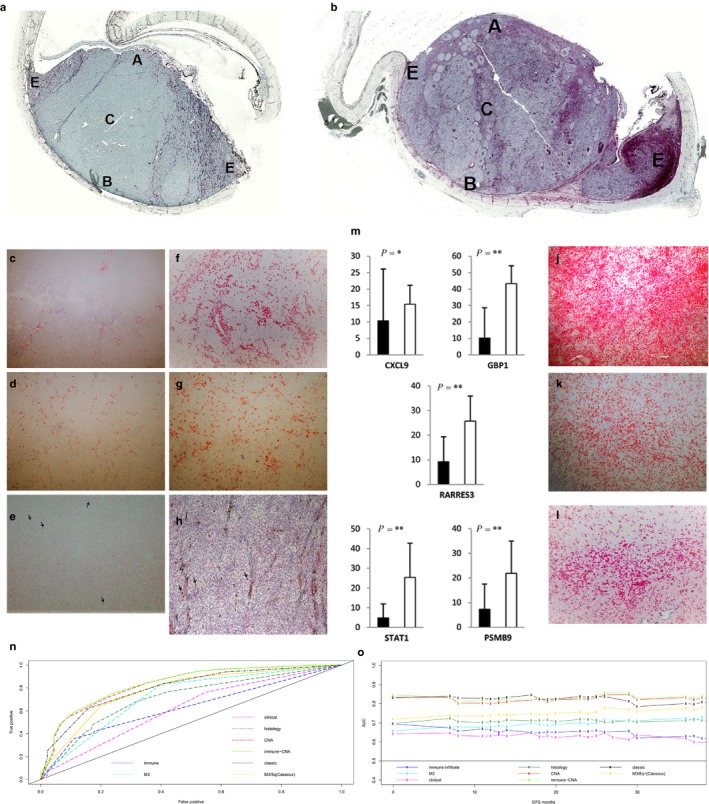
Immune signature defined by immunohistochemistry and ROC curves. a‐l: Intra‐tumoral distribution of immune cells in immune infiltrate‐low (a) and immune infiltrate‐high (b) uveal melanomas: edges (E), base (B), center (C) and apex (A). HLA‐DRA^+^ (c,f,j) and CD163^+^ (d,g,k) macrophages and CD3^+^T‐lymphocytes (e,h, black arrows & l) in immune infiltrate‐low and high uveal melanomas (Original magnification: 50X and 100X). (m) Expression of significant “immune” genes in immune infiltrate‐high (n = 5, □) and immune infiltrate‐low (n = 16, ■) uveal melanomas (*P*‐value: *≤0.05, **<0.01). (n‐o) Time‐dependent ROC curves (n) and AUC plots (o) up to 36 months for DFS for Cox univariate and multivariate models

Immunohistochemistry was performed on 13/15 UM profiled by microarrays. The intra‐tumoral densities of HLA‐DRA^+^CD163^+^macrophages and CD3^+^CD8^+^T‐lymphocytes encompassed: both edges (E), base (B), center (C), and apex (A) (Figure 3a,b). In UM defined as immune gene‐low in microarray analysis, macrophages were present at a mild‐to‐moderate level (Figure 3c,d,f,g) and T‐cells were occasional or absent (Figure 3e,h). In the immune gene‐high UM, macrophages and T‐lymphocytes were present in higher numbers (Figure 3j,k,l). These findings permitted to develop a semi‐quantitative, IHC‐based “immune score” (Materials and Methods). The mean scores in immune gene‐high and immune gene‐low tumors were 16.9 and 3.9, respectively. A cut‐off of 14 (mean score in immune‐high:16.9 minus twice the SD:1.29) was applied to categorize melanomas into immune infiltrate‐high or immune infiltrate‐low by IHC corresponding to immune gene‐high and immune gene‐low, respectively.

#### 3.2.2 Application of the IHC immune score to additional uveal melanomas

This IHC‐based immune score was applied to 76 additional tumors, categorizing them to 14 immune infiltrate‐high (II‐H) and 62 immune infiltrate‐low (II‐L). This subdivision was validated by RT‐qPCR on 5/14 II‐H and 16/62 II‐L UM, using the same five genes as previously applied. Expression was significantly higher in II‐H than in II‐L (*P* = 0.05‐0.002, Mann‐Whitney test, Figure 3m).

### 3.3 Validation of the IFN‐gamma signature by IHC

GBP1 was selected for validation for two reasons: it was the gene with highest fold difference in the immune signature and its transcription is strongly induced by IFNγ. GBP1 protein was detected mainly in tumor cell membrane and cytoplasm (Figure S2a,b), but also in some immune cells. The GBP1‐labeling index (Supplementary methods) was 18.6% in immune infiltrate‐high (SD:12.6) and 9.9% in immune infiltrate‐low (SD:8.9) UM (*P* = 0.03, Mann‐Whitney test, Figure S2c).

### 3.4 Immune signature compared to cancer germline and melanocyte differentiation genes

This comparison was performed as it is well established that cancer germline and melanocyte differentiation genes can elicit immune response in tumors.^11^ However, no statistically significant correlation was found between the expression of the latter genes and either, immune gene‐high or immune gene‐low tumors (Table S5).

### 3.5 Immune signature and copy number alterations

Copy number alterations (CNA) by MLPA was analyzable for 84/89 tumors. The alterations observed were as follows: Monosomy 3 (M3) in 48/84 (57.1%), 8q gain (8q+) in 41/84 (48.8%), isochromosome 8q (i8q) in 16/84 (19%), 6p gain (6p+) in 27/84 (32.1%) tumors, respectively. Loss of NBL1 (ΔNBL1) occurred either separately in 22/84 (26.2%) or as part of 1p loss in 18/84 (21.4%) tumors, respectively. Loss of LZTS1 (ΔLZTS1) occurred either separately in 19/84 (22.6%) or as part of 8p loss in 22/84 (26.2%) tumors, respectively (Figure S3).

In immune infiltrate‐high group, M3 was present in 17/19 (89.5%) tumors, with 16/17 (94.1%) also having 8q+, which in some cases was part of i8q (12/16, 75%) or 8 gain (8+; 3/16, 18.8%). In immune infiltrate‐low group, M3 was present in 31/65 tumors (47.7%) with concomitant 8q+ in 18/31 (58.1%) tumors, which in some cases was part of i8q (3/18, 16.7%) or 8+ (6/18, 33.3%). The immune infiltrate‐low tumors without M3 had i6p (12/34, 35.3%), ΔNBL1 (20/34, 58.8%) and ΔLZTS1 (18/34, 52.9%) (Figure S3).

### 3.6 Statistical analyses

#### 3.6.1 Survival analysis

The survival analysis was for DFS.

##### Univariate analysis

In Cox univariate analysis, the significant clinical variables were tumor stage and extrascleral extension. Histologically, they were characterized by epithelioid component, mitoses, necrosis, and immune cell infiltration. As copy number alterations (CNA), they included monosomy 3, isochromosome 8q, 8q gain (8q+), and 8p loss (Table S6, Figure S4a‐e). When looking individually at genes analyzed by MLPA, the majority of genes present on chromosomes 3 and 8q was significantly associated with outcome, as well as *NRG1*on 8p. The genes on chromosomes 1p and 6 were not significant (Table S7).

##### Multivariate analysis

Six multivariate models were constructed with the intention of comparing their performances in predicting the risk of metastases. The “classic model” was the conventional multivariate model derived from significant variables from univariate analysis (Supplementary methods). Of the others, four (clinical, histology, MLPA‐probe, CNA) were based on a single class of variables and one (immune‐CNA) on pooled class of variables. The reason for these latter models was to see if use of clinical data alone mitigates the need for genetic parameters that are expensive, and require invasive procedures.

The significant variables in classic, clinical, histology, MLPA‐probe, CNA, and immune‐CNA models are shown in Table 1, Tables S7 and S8.

**Table 1:** Cox multivariate analyses for the duration of DFS

Final model	HR (95% CI)	*P*‐value
Classic		
Mitoses	3.5 (1.4‐8.3)	0.005*
Extrascleral extension	8 (2.5‐25.2)	<0.001*
M3	3.1 (1.1‐9.1)	0.04*
Isochromosome 8q	4.2 (1.7‐10.3)	0.002*
Clinical		
Stage	2.5 (1.1‐5.8)	0.03*
Extrascleral extension	3.2 (1.2‐8.3)	0.02*
Histology		
Epithelioid component	2.1 (1.0‐4.1)	0.04*
Mitotic activity	2.9 (1.3‐6.5)	0.008*
Necrosis	2.1 (1.0‐4.6)	0.06
CNA		
M3	11.0 (3.4‐35.7)	<0.001*
8q+	3.9 (1.6‐9.2)	0.002*
*LZTS1* deletion	5.3 (2.2‐12.8)	<0.001*
*NBL1* deletion	5.3 (2.2‐13.0)	<0.001*
Immune‐CNA		
M3	9.2 (2.7‐31.1)	<0.001*
8q+	3.6 (1.5‐8.7)	0.005*
*LZTS1* deletion	4.6 (1.8‐11.6)	0.001*
*NBL1* deletion	5.8 (2.3‐14.8)	<0.001*
Immune infiltrate^a^	1.6 (0.7‐3.7)	0.2

a Immune forced into the model.

* *P* ≤ 0.05 considered significant.

The MLPA‐probe model was constructed to identify novel, gene‐based copy number changes that may be of significance, besides the conventional CNA. This led to the identification of deletion of *LZTS1*(Δ*LZTS1*) and *NBL1* (Δ*NBL1*) as novel variables (Table S7).

The immune‐CNA model, based on pooled classes of variables was constructed to understand the relation between immune cell infiltrate and various copy number alterations. The immune cell infiltration failed to retain its significance (*P* = 0.2, Table 1).

#### 3.6.2 Predictive accuracies of Cox models

The highest area under the curve (AUC) at 36 months for univariate models was for: M3 (0.725), 8q+ (0.711) and immune infiltrate (0.628) (Table S9). Among the multivariate models, it was immune‐CNA (0.837), CNA (0.832) and classic model (0.801) (Figure 3n). A comparable ranking was observed for integrated AUC (C^T^) until 36 months (Figure 3o). An already published model 4 based on M3 and 8q + applied to our series had AUC of 0.778 at 36 months (Table S9).

#### 3.6.3 Risk‐groups based on multivariate models

CNA, immune‐CNA, and classic models had the highest AUC. Three‐tier and four‐tier stratifications were performed for the former two (Supplementary methods/Table M3) and also for the published M3/8q+ (Cassoux) model.

##### CNA model

###### Tertile‐based risk‐groups

**Figure 4:**
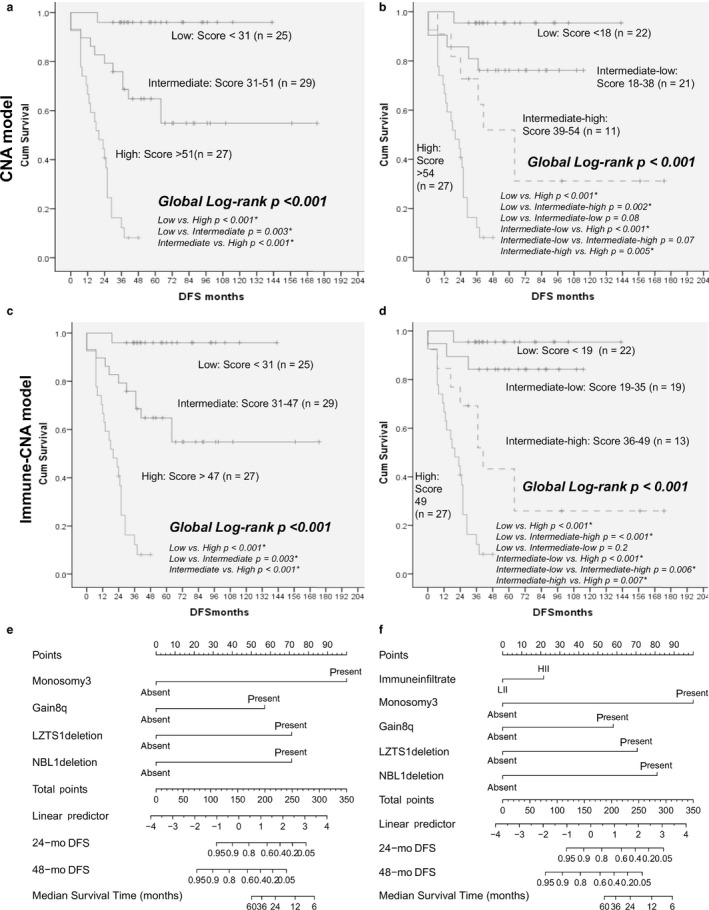
Kaplan‐Meier plots, cross‐validation and nomograms. Kaplan‐Meier plots for DFS for tertile‐based and quartile‐based risk‐groups in CNA model (a,b) and immune‐CNA (c,d) models. Nomograms for prediction of DFS based on CNA (e) and immune‐CNA (f) models

The median DFS in the high‐risk group was 20 months, but DFS was not reached in the intermediate and low‐risk groups (global log‐rank test *P* < 0.001). The adjusted (Benjamini‐Hochberg) log‐rank *P*‐values for pair‐wise comparisons between the three risk‐groups were all significant (Figure 4a). Metastases were developed by 24/27 (88.9%) patients in the high, 12/29 (41.4%) in the intermediate and 1/25 (4%) in the low‐risk groups, respectively. High‐risk group tumors displayed at least three CNA, intermediate two, except one tumor (three), and low‐risk group had no or one CNA (Figure S5a).

###### Quartile‐based risk‐groups

Kaplan‐Meier plots indicated statistical differences between the groups (global log‐rank test *P* < 0.001, Figure 4b). The intermediate group of tertile stratification was split into intermediate‐high and intermediate‐low. The adjusted (Benjamini‐Hochberg) log‐rank *P*‐values for pair‐wise comparisons were significant for all groups, except low‐risk vs intermediate‐low (*P* = 0.08) and intermediate‐low vs intermediate‐high (*P* = 0.07, Figure 4b), which showed a trend of significance.

For the intermediate‐high group, median DFS was 64 months; 7/11 patients (63.6%) developed metastases and M3+/Δ*NBL1*was the most frequent alteration (9/11, 81.8%). For the intermediate‐low group, median DFS was not reached and 5/21 patients (23.8%) developed metastases (Figure S5B). The low‐risk groups of both stratifications remained similar, with the exception of three tumors with only M3+ that were now included in intermediate‐low risk group.

##### M3/8q+ Cassoux model

The series was also tested according to the published model, which is based on the status of chromosome 3 and 8q+.^4^ The KM plots indicated differences between the risk groups tested (log‐rank test *P* < 0.001, Figure S4f).

##### Immune‐CNA model

###### Tertile‐based risk‐groups

There were significant differences in DFS between the three groups (global log‐rank test *P* < 0.001, Figure 4c). The adjusted (Benjamini‐Hochberg) log‐rank *P*‐values for pair‐wise comparisons between the three groups were all significant and same as for the tertile‐based CNA model.

Out of the 19 immune infiltrate‐high UM, 14 stratified to high (73.7%), four to intermediate (21%) and one to low‐risk groups (5.3%). For the immune infiltrate‐low tumors, 13/62 (21%) were found in the high‐risk group, 25/62 (40.3%) in the intermediate and 24/62 (38.7%) in the low ones. The median DFS and the CNA associated with the risk‐groups were the same as for the CNA model (Figure S5c).

###### Quartile‐based risk‐groups

The intermediate risk‐group was divided into intermediate‐high and intermediate‐low. The adjusted (Benjamini‐Hochberg) log‐rank *P*‐values for pair‐wise comparisons between the four groups were significant for all, except low‐risk vs intermediate‐low (*P* = 0.2, Figure 4d).

The intermediate‐high group (n = 13) included 3/19 II‐H (15.8%) and 10/62 II‐L (16.1%), median DFS was 41 months and 9/13 patients (69.2%) developed metastases. In intermediate‐low tumors (n = 19), there were 1/19 II‐H (5.3%) and 18/62 II‐L tumors (29%), median DFS was not reached, and 3/19 (15.8%) patients developed metastases (Figure S5d).

###### Impact of immune cell infiltration

In each risk category, the prognostic impact of immune cell infiltration was compared between tumors with identical CNA. In the high‐risk group, whatever the degree of immune response, the DFS was comparable (log‐rank test *P* = 0.75, Figure S6a). In the intermediate‐risk group, DFS was shorter in II‐H when compared to immune‐low (log‐rank test *P* = 0.04, Figure S6b). In the low‐risk group, the analysis was not feasible, as there was only one II‐H tumor.

#### 3.6.4 Validation of multivariate Cox models

##### Cross‐validation

A leave‐one‐out cross‐validation using the study dataset was performed on CNA and immune‐CNA models (Figure S7a‐d). The misclassification error rates were: 1.2% and 1.2% for the three‐group stratification, and 24.7% and 8.6% for the four‐group stratification, respectively.

##### External validation of multivariate Cox models

The TCGA data from uveal melanomas were used for external validation of CNA and immune‐CNA models.^9^ The TCGA tumors were classified into three risk groups based on the above described CNA model comprising M3, 8q gain, LZTS1, and NBL1 deletions. The KM plot showed significant differences between the three risk‐groups (global log‐rank *P* < 0.001, Figure S8a). The adjusted (Benjamini‐Hochberg) log‐rank *P*‐values for pair‐wise comparisons between the three risk‐groups were all significant (Figure S8a). The four risk‐group CNA model could not be validated as there was only one tumor in the intermediate‐high risk group.

The three risk‐group immune‐CNA model could also be validated (global log‐rank *P* < 0.001, Figure S8b). Likewise, the four risk‐group immune‐CNA model too showed significant survival differences between the four risk‐groups (global log‐rank *P* < 0.001, Figure S8c). The adjusted (Benjamini‐Hochberg) log‐rank *P*‐values for pair‐wise comparisons between the four groups were not significant for low‐risk vs intermediate‐low, intermediate‐low vs intermediate‐high and intermediate‐high vs high (Figure S8c).

In addition to validating the present CNA model comprising M3, 8q gain, LZTS1, and NBL1 deletions on the TCGA data, the mutations associated with the tumors in high, intermediate and low‐risk groups were assessed. The majority of tumors, 29/35 (82.6%) in the high‐risk group showed BAP1 mutations. These tumors had M3/8q gain with either 8p loss or LZTS1 deletion or NBL1 deletion. The intermediate risk‐group melanomas had either BAP1 (4/13, 30.7%) or SF3B1 (5/13, 38.5%) mutations. The low‐risk group tumors had either SF3B1 (10/32, 31.2%) or EIF1AX (10/32, 31.2%) mutations. All the tumors with EIF1AX mutations lacked M3, 8q gain, LZTS1 or NBL1 deletions.

##### External validation of the impact of immune cell infiltration

The TCGA uveal melanomas were classified into immune‐high and immune‐low categories based on the combined tumor‐infiltrating‐lymphocyte density and tumor‐associated‐macrophage density (Supplementary Methods, Table M4). The impact of immune infiltration in uveal melanomas with high‐risk CNA groups was tested. KM plots comparing time to metastasis between immune‐high and immune‐low groups (log‐rank *P* = 0.98, Figure S8d), showed no significant difference between the two groups. The impact of immune cell infiltration in the intermediate‐risk CNA groups could not be tested as there was only one immune‐high uveal melanoma.

#### 3.6.5 Nomogram

Nomograms were constructed for CNA and immune‐CNA models for DFS prediction (Figure 4e,f). In the CNA model‐based nomogram, the DFS probabilities at 24 and 48 months were 40% and 10%, respectively, for the high‐risk group.

The risk of developing metastasis within 48 months of diagnosis from this CNA‐based nomogram was compared to those from PRiMeUM, a web‐based tool.^12^ PRiMeUM predicts the metastatic risk based on alterations in chromosomes 1, 3, 6, and 8, patient age, tumor location, diameter, and thickness. Despite this difference, pair‐wise *t* test comparing the predictions from the present CNA model and PRiMeUM did not reveal any significant difference (*P* = 0.28).

## 4 DISCUSSION

Immune cell infiltration in solid tumors is usually associated with a transcriptomic signature that includes genes regulated by interferon‐gamma (IFNγ), as illustrated in breast, colorectal, and ovarian carcinomas, as well as in cutaneous melanomas.^13^, ^14^, ^15^, ^16^ Similarly, gene expression profiling allowed us to identify a subset of uveal melanomas (UM) that expressed genes of the IFNγ/STAT1‐IRF1 pathway. Observation of CD3^+^CD8^+^ T lymphocyte and HLA‐DRA^+^CD163^+^ macrophage infiltration in these tumors, and demonstration of GBP1 expression in tumor cells also attested to the activation of this pathway.

Besides the STAT1‐IRF1 pathway, the transcriptomic signature included other immune‐related genes, such as those encoding lymphocyte and macrophage attracting chemokines, including *CCL4*, *CCL5*, *CXCL9,* and *CXCL10*. The signature also comprised molecules of the cytolytic pathway (*CD8A*, *PRF*, *GZMB*) and adhesion molecules (*SIGLEC1*, *LGMN*). These results indicate an IFNγ‐associated T‐helper 1 (Th1) orientation of the T cell infiltrate present in some UM. The identified signature correlates with the recently reported one from TCGA project.^9^

It is intriguing that even though uveal melanomas and other cancers have similar IFNγ‐induced immunological profiles, there is an opposing correlation with clinical outcome. The degree of T‐lymphocyte infiltration is associated with better survival in most solid tumors,^17^ but not in UM.^18^, ^19^ Our data confirm this observation.

This differential impact of immune response may be related to another player in immune response, the tumor‐associated macrophages (TAMs). For the latter, our results underscore the literature; in uveal melanomas, presence of TAMs is associated with worse prognosis,^20^ just like in most cancers.^21^ Yet, we lack a molecular understanding of these differences.

In this era of molecular medicine and personalized therapy, better stratification of UM is of paramount importance, as there is no adjuvant therapy for these tumors following surgery or radiotherapy. An additional motivation for better stratification of UM is based on the suboptimal efficiency of monosomy 3 (M3), which has for long been considered as the best biomarker for uveal melanoma stratification.^3^ A gene expression profile (GEP)‐based test identified two better subclasses of tumors: the class 1‐GEP, with genes associated to *EIF1AX* mutations, and the class 2‐GEP, linked to *BAP1*and a poorer outcome.^3^, ^22^

To understand better the relative value of biomarkers in uveal melanoma prognostication, we designed five different Cox multivariate models: clinical, histological, CNA, immune‐CNA and classic. Their performances were compared to published DNA biomarkers, M3, and M3/8q+,^2^, ^4^ using ROC curves.^6^ All our multivariate models, except the clinical one, performed better than M3 only.

Among our models, the CNA, immune‐CNA, and classic models had the best performance and were more accurate in predicting DFS in uveal melanomas than the conventional binary grouping. Stratification using the CNA and immune‐CNA models indicated the presence of three/four risk‐groups in uveal melanomas. Moreover, the CNA and immune‐CNA models could be validated on an independent, external dataset from TCGA.^9^ These results reinforce the recently published data from TCGA, describing four molecular subgroups in uveal melanomas based on somatic copy number alterations (SCNA), transcriptional profile and methylation patterns,^9^ and advocates their use in future clinical trials.

The CNA model was based on four DNA alterations: M3, 8q+, *LZTS1* deletion (Δ*LZTS1*), and *NBL1*deletion (Δ*NBL1*). It defines risk‐groups according to the quantity of genetic anomalies: three or more, in the high‐risk group that comprises of patients having 90% probability of metastasis at 3 years. None, in the low‐risk group, that includes patients with 90% probability of DFS at 10 years. This is understandable, as aneuploidy correlates with prognosis in cancer.^23^, ^24^ In addition, this reinforces the somatic CNA‐based groups in the TCGA data, where the low‐risk group showed the least aneuploidy.^9^ Moreover, in view of its performance, robustness, low‐cost, applicability on fine‐needle aspiration samples and potential use for circulating tumor DNA, the CNA model appears most appropriate for clinical use.

In this model, the addition of *LZTS1* and *NBL1*status refined the prognostic significance of a published model based on two anomalies: M3 and 8q+.^4^ Indeed, combined M3+/8q + was observed in UM of both intermediate and high‐risk groups, but only in the latter when associated with Δ*LZTS1* and/or Δ*NBL1*. This is expected, as Δ*LZTS1*is associated with metastatic potential in uveal melanomas^25^ and *NBL1* to tumor progression in cancers.^26^, ^27^

Although somatic CNA are dominant features in cancers,^28^, ^29^ there is a paucity of data regarding their association with immune cell infiltration or the expression of immune‐related genes. In breast cancer, such immune profiles have been associated with CNA‐devoid tumors,^28^ in head and neck cancers with 13q loss^30^ and in uveal melanomas with M3.^9^, ^31^ In our series too, high degree of immune infiltration was associated with M3 (89.5%); yet, only 35% of UM with M3 had high immune infiltration compared to the rest of the tumors (65%) in which immune infiltration was low. To the best of our knowledge, there are no prognostic models in cancer incorporating copy number aberrations and immune infiltration. This is surprising, considering their importance as biomarkers in general.

Our immune‐CNA model showed occurrence of immune infiltrate‐high UM through all risk groups. It was more frequent in high‐risk group (73.7%) than in intermediate (21%) and low‐(5.3%) risk groups, but was not an independent prognostic factor from the studied CNA. In contrast, in colorectal cancer the survival benefit of lymphocytic infiltration is an independent factor.^32^

In addition, there was no survival difference between immune infiltrate‐high and immune infiltrate‐low UM (*P* = 0.75) in the high‐risk group of immune‐CNA model, indicating a neutral effect of the T‐cell infiltration in this risk‐group. This finding was also validated on an independent, external dataset from TCGA.^9^ This may appear contradictory to the published data, in which immune response is associated to a negative outcome.^19^ However, this is not the case, as the impact of T‐cell infiltration has neither been compared with other biomarkers, nor analyzed with respect to three risk groups in the literature.

In the intermediate‐risk group, the outcome of immune infiltrate‐high UM was worse than in immune infiltrate‐low ones (*P* = 0.04). The reason for this is unclear. If confirmed on independent series, this would raise an important question from a therapeutic point of view ‐ whether immune component in uveal melanomas needs to be targeted in a different manner according to the various risk groups.

The caveats of this study are: relatively small sample size, bias toward large sized tumors, and use of MLPA technique to assess copy number alterations. The use of the latter technique and a prognostic model based on the same may be questioned in this era of whole‐exome or targeted sequencing, where the mutational profiles and the copy number alterations can be derived from the sequencing data. It is true that the use of whole‐exome or targeted sequencing has led to the identification of *BAP1*, *SF3B1,* and *EIF1AX* (BSE) mutations, each of which is associated with a specific set of copy number alterations.^9^, ^33^, ^34^ However, the use of next‐generation sequencing (NGS) requires adequate infrastructure and bioinformatic support. Moreover, certain complex genetic alterations in BSE mutations can make this task more difficult. For example, BAP1 mutations can frequently be missed when they comprise large insertions/deletions, intronic/splice site alterations and other complex rearrangements. In order to ensure that these alterations are identified, it is necessary to use additional bioinformatic tools and customize the pipeline.^33^ Even though they have tremendous potential, the data and cost burden remain substantial, despite a large reduction in cost over the years. MLPA, on the other hand is simpler, both technically and analysis‐wise. Therefore, MLPA still has a role in risk‐stratification of uveal melanoma patients. It may be taken over by NGS in the near future, but for now, it is still a useful technique for uveal melanoma prognostication. Hence, the present CNA model and CNA‐based nomogram are useful adjuncts to other prognostic parameters.

In conclusion, the IFNγ/STAT1‐IRF1 signature as a component of the immune response in uveal melanomas has been validated in vivo. Comparison of immune cell infiltration as a biomarker to other conventional markers established the superiority of a three risk group copy number alteration model for uveal melanoma prognostication. It also unraveled two hitherto previously unreported features. First, the differential prognostic influence of immune cell infiltration according to risk groups, suggesting that the impact of immune cells on survival is copy number driven. Second, the demonstration that a large proportion of immune‐high uveal melanomas carry high‐risk DNA alterations that might explain their poor clinical outcome.

## CONFLICT OF INTEREST

The authors declare no potential conflicts of interest.
