# Supplementary material for: DNA alteration‐based classification of uveal melanoma gives better prognostic stratification than immune infiltration, which has a neutral effect in high‐risk group
Source: Cancer Med. 2019 Apr 25;8(6):3036–46. doi: 10.1002/cam4.2122 (PMC6558590; doi:10.1002/cam4.2122)
Supplement: Supplementary file 1 [file CAM4-8-3036-s001.pdf]

Supplementary Figures

(S1a)

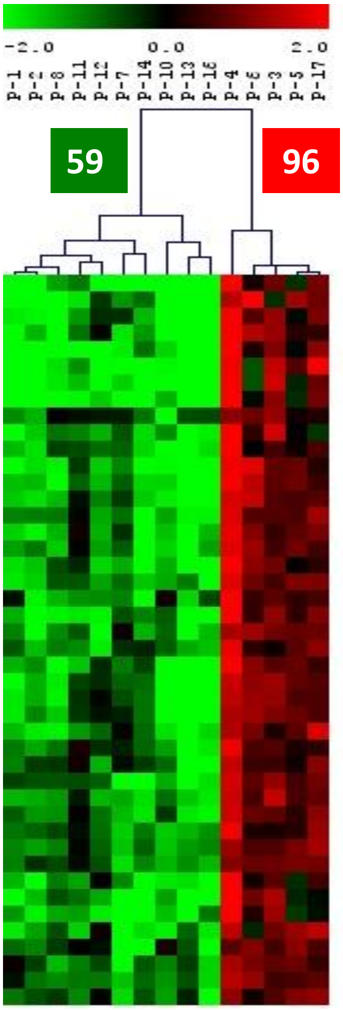

(S1b)

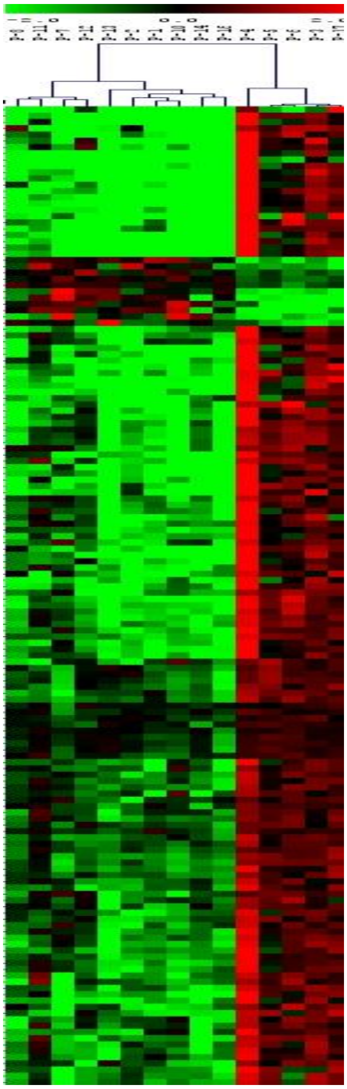

Figure S1. Microarray analyses

S1a. Heat map of 44 probe sets isolated from t-test performed on the 1500 probe sets with the highest standard deviation. Node values (59 and 96) obtained by bootstrapping (100 iterations).

S1b. Heat map of 156 probe sets isolated from T-test performed on all immune response genes

(red and green indicate over and underexpression, respectively).

(S2a)

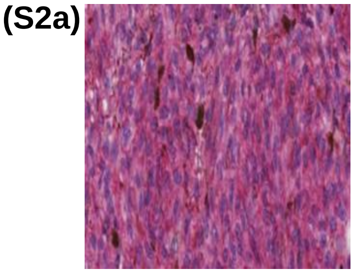

(S2b)

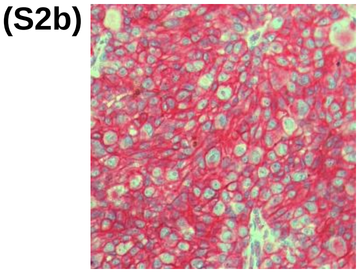

(S2c)

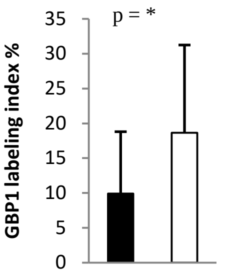

Figure S2. GBP1 immunohistochemistry

S2a and S2b. Detection of GBP1 protein in immune infiltrate-low (S2a) and immune infiltrate-high (S2b) uveal melanoma.

S2c. Comparison of GBP1-labeling indices in immune infiltrate-high and immune infiltrate-low uveal melanomas.

(p-value: * ≤ 0.05)

(S3)

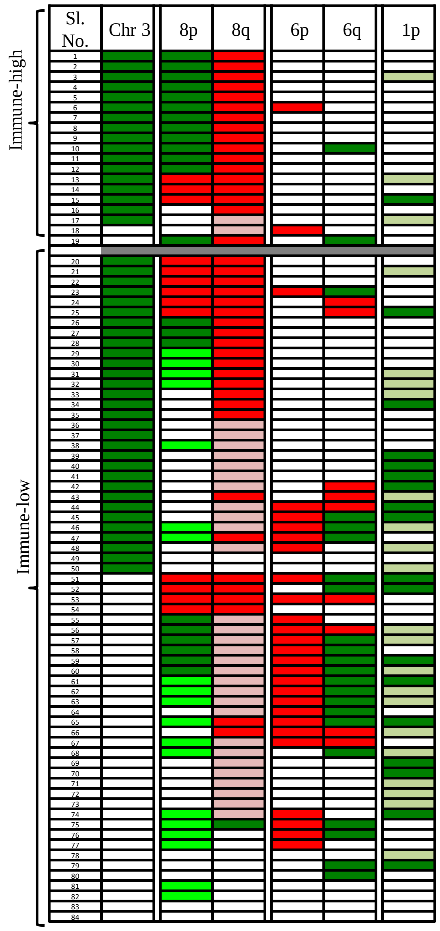

Figure S3. Schematic representation of copy number alterations in immune-high and immune-low melanomas (n = 84).
(■ Loss ■ Gain ■ *MYC* gain ■ *LZTS1* deletion ■ *NBL1* deletion)

(S4a)

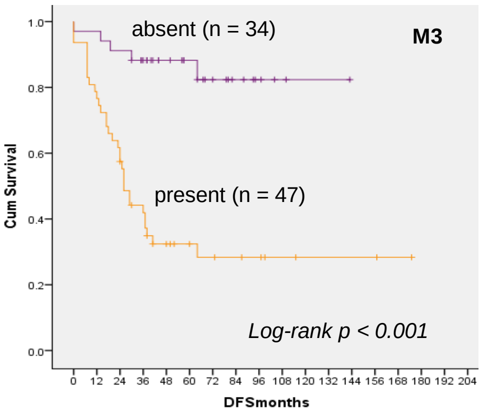

(S4b)

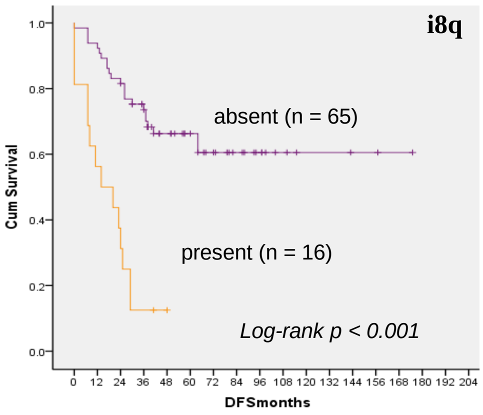

(S4c)

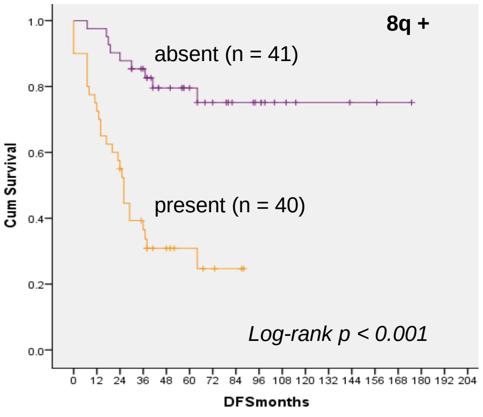

(S4d)

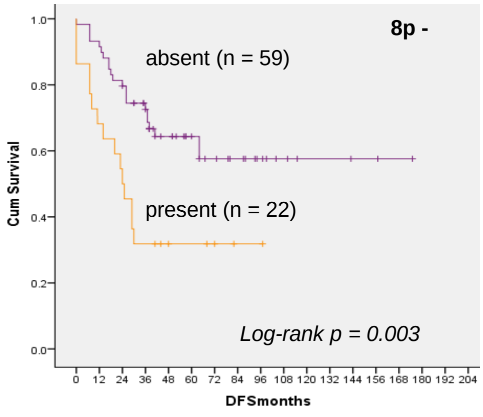

(S4e)

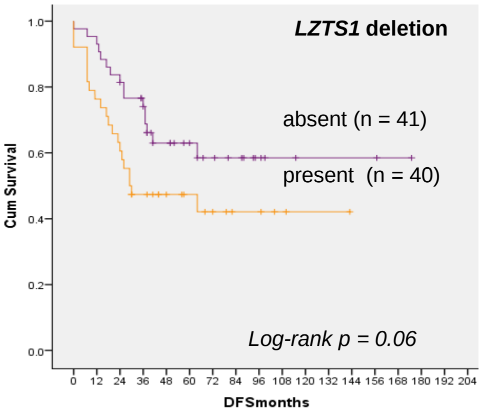

(S4f)

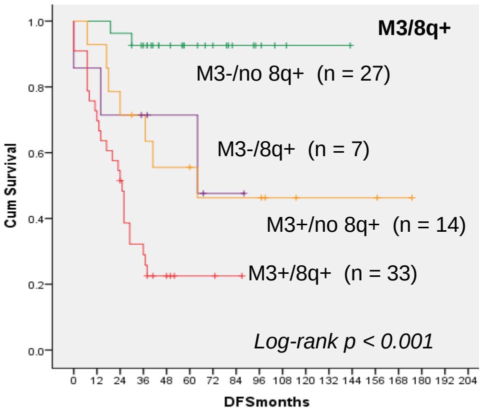

Figure S4. Kaplan-Meier plots for the duration of DFS in uveal melanomas with different chromosomal aberrations

(S5a)

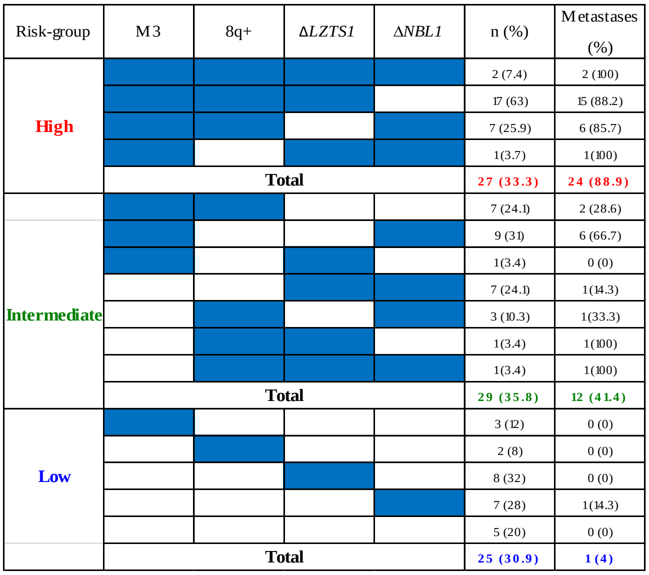

(S5b)

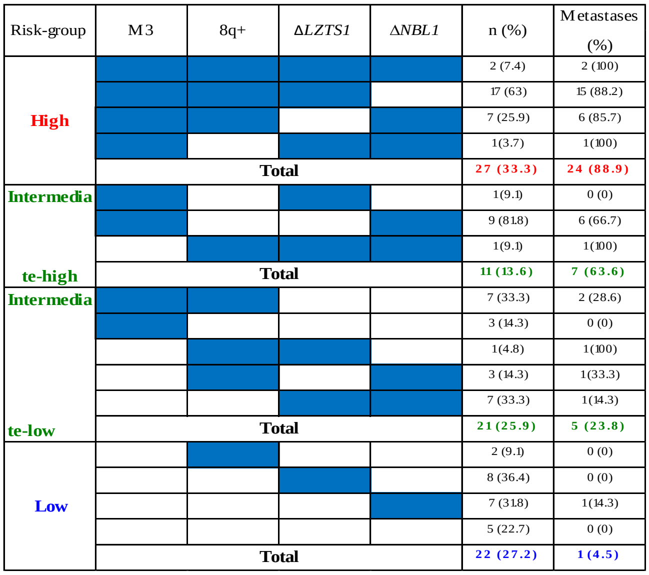

(S5c)

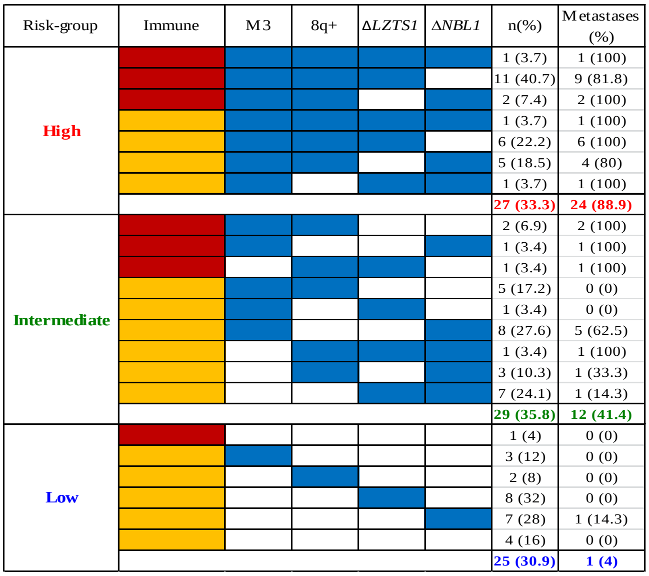

(S5d)

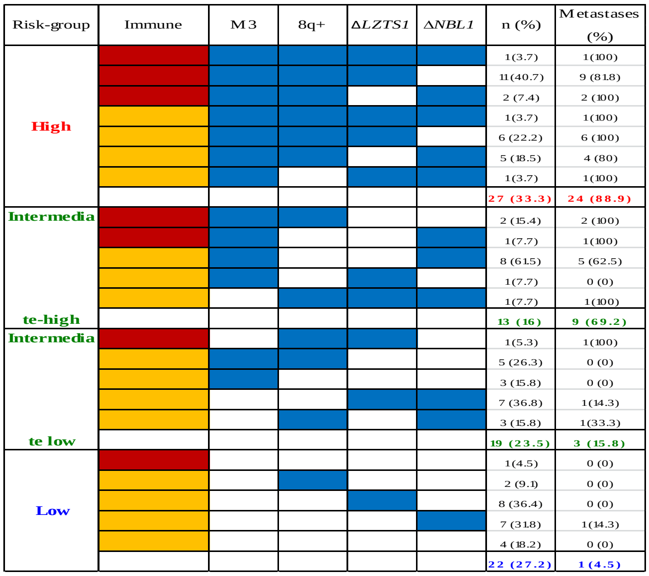

Figure S5. Schematic representation of alterations in CNA and immune-CNA models

S5a and S5b: Alterations for tertile-based (S5a) and quartile-based (S5b) risk-groups in CNA-model.
S5c and S5d: Alterations for tertile-based (S5c) and quartile-based (S5d) risk-groups in immune-CNA model.
In the tertile-based, the most common genetic profile in: high-risk group was M3+/8q+/Δ*LZTS1* (19/27, 70.4%; 12 immune-high and 7 immune-low); intermediate risk-group was M3+/Δ*NBL1* (9/29, 31%; one immune-high and eight immune-low); and low-risk group were Δ*LZTS1* (8/25, 32%) and Δ*NBL1* (7/25, 28%), both in immune-low tumors only. In the quartile-based, the most frequent alteration in intermediate-high group was M3+/Δ*NBL1* (9/13, 69.2%) and in intermediate-low was co-deletion of Δ*LZTS1*/Δ*NBL1* (7/19, 36.8%).
(CNA-present CNA-absent immune-high immune-low)

(S6a)

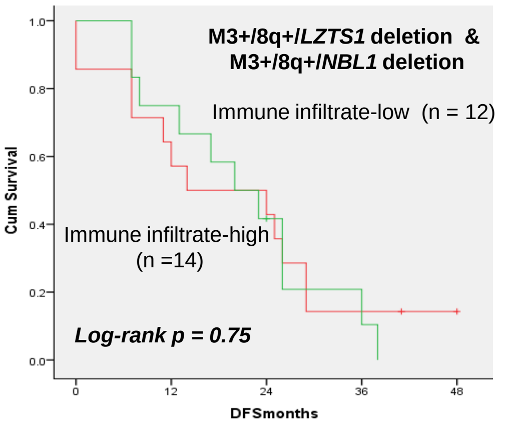

(S6b)

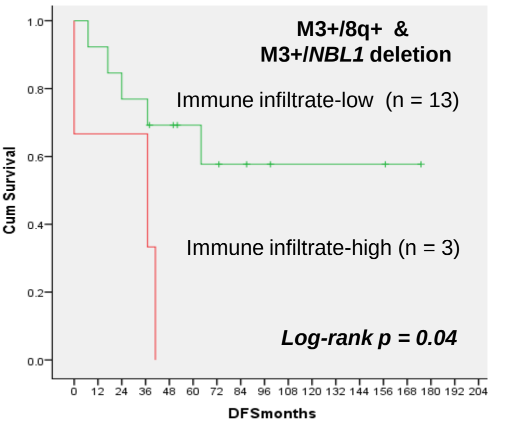

Figure S6. Prognostic relevance of immune infiltrate in uveal melanomas with similar CNA

S6a: Kaplan-Meier plots for the duration of DFS between immune infiltrate-high and immune infiltrate-low tumors in high-risk CNA

S6b: Kaplan-Meier plots for the duration of DFS between immune-high and immune-low tumors in intermediate-risk CNA

(S7a) CNA model (S7b)

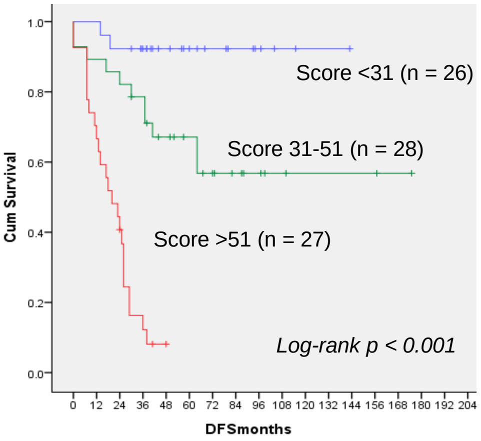

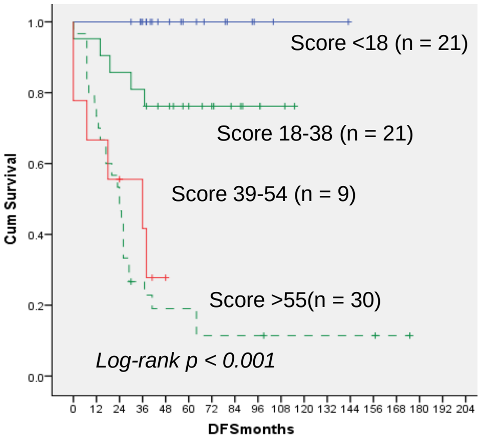

(S7c) Immune-CNA model (S7d)

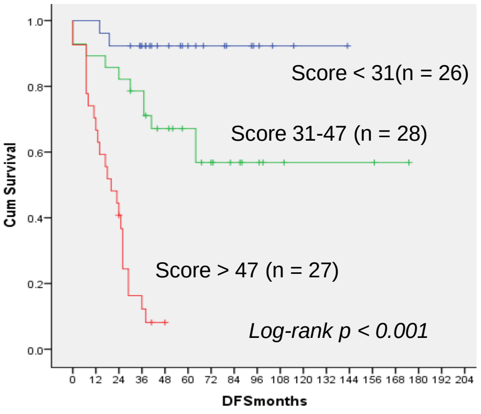

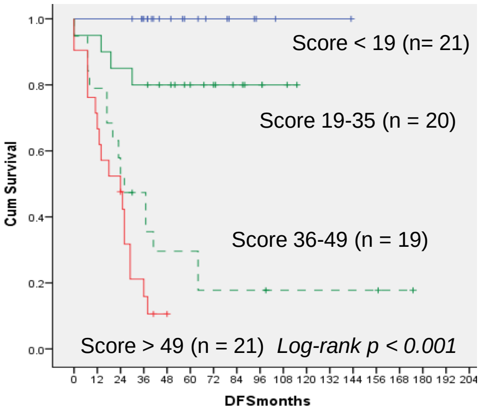

Figure S7. Prognostic groups based on cross-validated multivariate models
S7a and S7b: Kaplan-Meier plots for the duration of DFS based on cross-validated tertile-based (S7a) and quartile-based (S7b) risk-groups in CNA model
S7c and S7d: Kaplan-Meier plots for the duration of DFS based on cross-validated tertile-based (S7c) and quartile-based (S7d) risk-groups in immune-CNA model

(S8a)

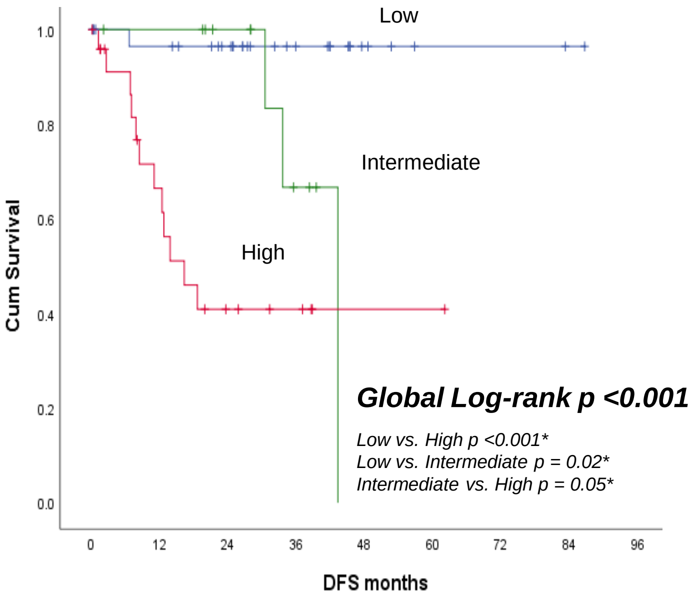

(S8b)

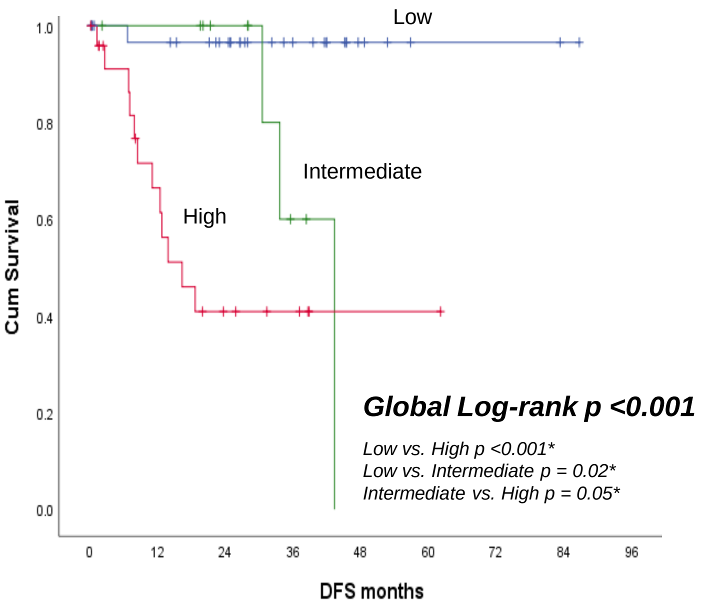

(S8c)

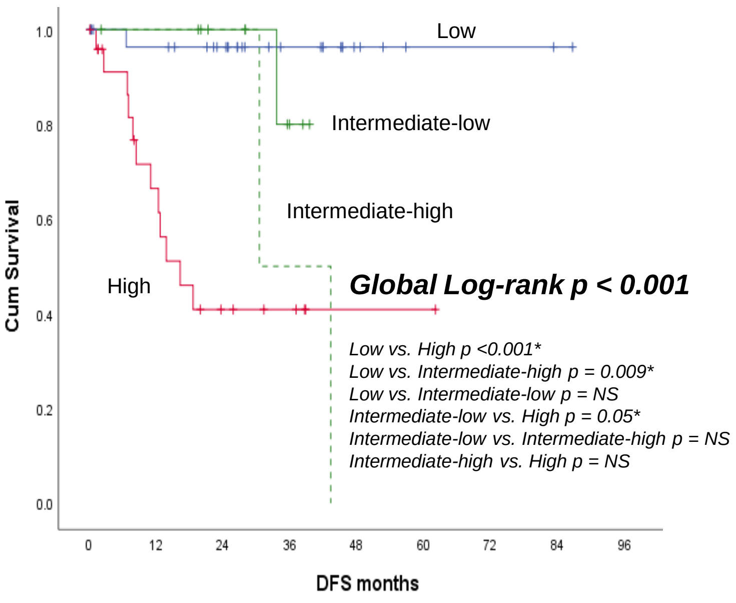

(S8d)

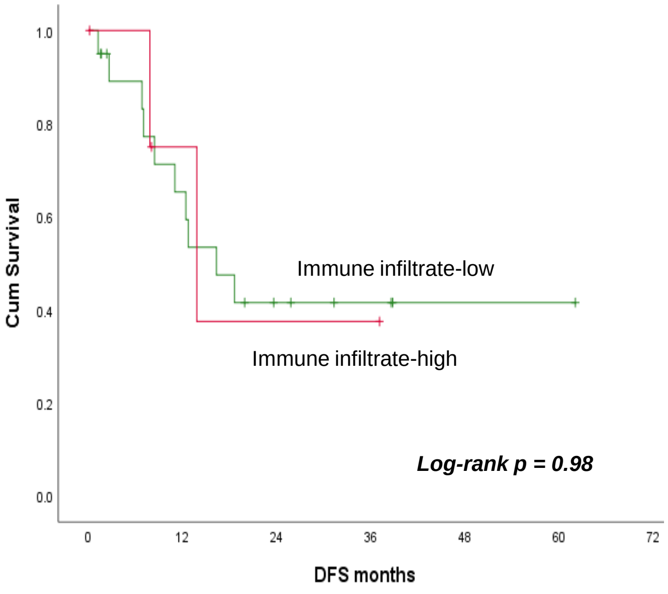

Figure S8. External validation of CNA, immune-CNA and the impact of immune cell infiltration in TCGA data

S8a: Kaplan-Meier plots for the duration of DFS between high, intermediate and low-risk groups of the CNA model

S8b: Kaplan-Meier plots for the duration of DFS between high, intermediate and low-risk groups of the immune-CNA model

S8c: Kaplan-Meier plots for the duration of DFS between high, intermediate-high, intermediate-low and low-risk groups of the immune-CNA model

S8d: Kaplan-Meier plots for the duration of DFS between immune infiltrate-high and immune infiltrate-low tumors with high-risk CNA

(* p-value significant, NS: not significant)
