# Supplementary material for: DNA alteration‐based classification of uveal melanoma gives better prognostic stratification than immune infiltration, which has a neutral effect in high‐risk group
Source: Cancer Med. 2019 Apr 25;8(6):3036–46. doi: 10.1002/cam4.2122 (PMC6558590; doi:10.1002/cam4.2122)
Supplement: Supplementary file 2 [file CAM4-8-3036-s002.pdf]

Table S1. *Baseline details for tumor series*

Clinical features		Valid N	Not available
**Age at diagnosis(years)**		89	0
Mean(SD†)	64.7(13.5)		
Median(Range)	67(35-90)		
upto 60 years	32(36%)		
> 60 years	57(64%)		
**Gender**		89	0
Female	43(48.3%)		
Male	46(51.7%)		
**Stage**		78	11(12.4%)
IIA	6(7.7%)		
IIB	20(25.6%)		
IIIA	17(21.8%)		
IIIB	30(38.5%)		
IIIC	1(1.3%)		
IV	4(5.1%)		
**T category**		77	12(13.5%)
T1c	1(1.3%)		
T2a	5(6.5%)		
T2b	1(1.3%)		
T3a	20(25.9%)		
T3b	10(13%)		
T3c	2(2.6%)		
T4a	5(6.5%)		
T4b	31(40.3%)		
T4c	1(1.3%)		
T4d	1(1.3%)		
**Basal diameter (mm)**		77	12(13.5%)
Mean(SD)	17.8(3.1)		
Median(Range)	18(7-25)		
upto 15mm	16(20.8%)		
>15mm	61(79.2%)		
**Thickness(mm)**		84	5(5.6%)
Mean(SD)	10.5(3.1)		
Median(Range)	11.1(1.6-18)		
upto 8mm	16(19%)		
>8mm	68(81%)		
**Location**		89	0
Choroid	37(41.6%)		
Choroid & ciliary body	52(58.4%)		
**Retinal detachment**		81	8(9%)
absent	2(2.5%)		
present	79(97.5%)		
**Extrascleral extension**		89	0
absent	82(92.1%)		
present	7(7.9%)		
Histologic features			
**Epithelioid component (%)**		88	1(1.1%)
Mean(SD)	29.7(31.2)		
Median(Range)	20(0-100)		
upto 25%	51(58%)		
> 25%	37(42%)		
**Mitoses (40hpf)**		88	1(1.1%)
Mean(SD)	12.7(11)		
Median(Range)	11(0-54)		
upto 10/40hpf	43(48.9%)		
>10/40hpf	45(51.1%)		
**Pigment**		89	0
Mean(SD)	16.2(21.6)		
Median(Range)	7.5(0-100)		
upto 25%	69(77.5%)		
>25%	20(22.5%)		
**Necrosis**		89	0
absent	78(87.6%)		
present	11(12.4%)		

†SD: standard deviation

Table S2. *Overexpressed and underexpressed significant probe sets identified from HCL and immune gene analysis*

Probe sets overexpressed in immune gene-high tumors

HCL significant genes*				
Gene symbol	Probeset ID	Average expression		Fold change
		Immune gene-high	Immune gene-low	
GBP1	202269_x_at	1259.56	87.36	14.4
CCL5	204655_at	498.46	36.78	13.6
RARRES3	204070_at	1792.2	147.55	12.1
PSMB9	204279_at	1214.04	101.88	11.9
PRF1	214617_at	306.26	27.53	11.1
CD74	1567628_at	2516.48	299.4	8.4
FGL2	227265_at	628.62	76.89	8.2
C1QA	218232_at	2589.12	323	8.0
CCL4	204103_at	195.66	26.99	7.2
HLA-DQA1 /// HLA-I	212671_s_at	2340.42	333.85	7.0
HLA-DRA	210982_s_at	3599.94	518.12	6.9
C1QB	202953_at	2988.62	440.41	6.8
UBE2L6	201649_at	1520.18	224.7	6.8
GIMAP4	219243_at	324.84	51.61	6.3
FGL2	204834_at	363.92	60.81	6.0
CD27	206150_at	250.24	43.21	5.8
HLA-DPA1	211991_s_at	4409.98	768.5	5.7
GBP2	202748_at	409.06	72.98	5.6
C1QC	225353_s_at	3381.78	615.82	5.5
HLA-DRB1 /// HLA-I	215193_x_at	5288	990.45	5.3
IRF1	202531_at	408.84	79.3	5.2
SLC15A3	219593_at	529.42	104.63	5.1
APOL6	219716_at	305.32	60.83	5.0
HLA-G	211530_x_at	4790.08	958.17	5.0
MPEG1	226818_at	767.9	156.33	4.9
MPEG1	226841_at	394.5	81.08	4.9
APOL6	1557116_at	391.02	82.45	4.7
HLA-DRB1 /// HLA-I	209312_x_at	6458.72	1366.29	4.7
STAT1	200887_s_at	3630.14	784.04	4.6
HLA-DRB6	217362_x_at	511.58	113.56	4.5
HLA-DRB1 /// HLA-I	204670_x_at	5147.34	1174.07	4.4
APOL6	241869_at	333.38	83.13	4.0
HLA-E	200904_at	4159.3	1080.39	3.8
BATF2	228439_at	225.46	58.8	3.8
TRIM22	213293_s_at	950	252.46	3.8
PSMB10	202659_at	647.56	173.88	3.7
TAPBPL	218747_s_at	283.64	76.73	3.7
HLA-E	200905_x_at	7782.54	2237.12	3.5
HCLS1	202957_at	365.9	109.9	3.3
TAPBPL	218746_at	257.84	78.95	3.3
HLA-J	217436_x_at	3100.42	986.82	3.1
RNASE6	213566_at	405.34	136.83	3.0
FKBP11	219118_at	1039.04	360.55	2.9
FAM119A	235931_at	87.98	32.66	2.7
**From immune gene analysis***				
CXCL9	203915_at	1262.9	36.8	34.3
IDO1	210029_at	610.8	18.0	33.9
IL32	203828_s_at	191.0	6.2	30.9
IGHM	216491_x_at	263.3	9.5	27.8
NKG7	213915_at	283.9	12.3	23.1
GBP1	202270_at	859.6	37.3	23.1
GBP1	231577_s_at	1953.0	96.4	20.3
CCL5	1405_i_at	648.5	32.9	19.7
GABBR1 /// UBD	205890_s_at	609.9	33.6	18.1
TRBC1	211796_s_at	569.3	32.9	17.3
TRAC	209671_x_at	468.1	28.8	16.2
TRAC /// TRAJ17 /// 7	210972_x_at	617.4	38.5	16.0
IKZF3	227030_at	122.7	7.8	15.7
GBP1	202269_x_at	1259.6	87.4	14.4
CCL5	1555759_a_at	727.4	52.9	13.7
APOL1	209546_s_at	200.6	14.7	13.7
ADAMDEC1	206134_at	409.4	30.0	13.6

CCL5	204655_at	498.5	36.8	13.6
LCK	204891_s_at	127.0	10.5	12.1
STAT1	209969_s_at	693.5	57.9	12.0
PSMB9	204279_at	1214.0	101.9	11.9
CD8A	205758_at	277.4	24.6	11.3
PRF1	214617_at	306.3	27.5	11.1
LYZ	1555745_a_at	687.2	63.1	10.9
LAG3	206486_at	106.3	10.0	10.6
PTPRCAP	204960_at	91.1	8.7	10.4
NFATC2	226991_at	67.7	7.0	9.7
TRBC1	210915_x_at	486.1	51.8	9.4
CD2	205831_at	367.9	39.3	9.4
C1S	1555229_a_at	240.2	27.6	8.7
CD74	1567628_at	2516.5	299.4	8.4
ERAP1	214012_at	84.1	10.1	8.3
IL12RB1	1552584_at	214.1	25.8	8.3
CD38	205692_s_at	115.8	14.3	8.1
C1QA	218232_at	2589.1	323.0	8.0
AIF1	209901_x_at	329.5	41.2	8.0
IL21R	219971_at	28.3	3.6	8.0
INPP5D	203331_s_at	18.5	2.4	7.9
LCK	204890_s_at	71.5	9.4	7.6
SH2D1A	210116_at	56.5	7.7	7.4
CORO1A	209083_at	185.4	25.6	7.2
CCL4	204103_at	195.7	27.0	7.2
HLA-DQA1 /// HLA-I	212671_s_at	2340.4	333.9	7.0
SLAMF7	222838_at	192.6	27.6	7.0
HLA-DRA	210982_s_at	3599.9	518.1	6.9
GZMB	210164_at	100.6	14.5	6.9
C1QB	202953_at	2988.6	440.4	6.8
UBE2L6	201649_at	1520.2	224.7	6.8
CD3D	213539_at	236.7	36.3	6.5
THEMIS	1558971_at	21.8	3.7	5.9
CD27	206150_at	250.2	43.2	5.8
TRBC1	213193_x_at	532.1	92.4	5.8
HLA-DPA1	211991_s_at	4410.0	768.5	5.7
FYB	227266_s_at	199.8	35.6	5.6
GBP2	202748_at	409.1	73.0	5.6
C1QC	225353_s_at	3381.8	615.8	5.5
TLR8	229560_at	121.7	22.5	5.4
TAP1	202307_s_at	1536.4	284.2	5.4
HLA-DRB1 /// HLA-I	215193_x_at	5288.0	990.5	5.3
IRF1	202531_at	408.8	79.3	5.2
TNFSF10	202688_at	196.9	38.4	5.1
HLA-G	211530_x_at	4790.1	958.2	5.0
C1S	208747_s_at	843.3	170.3	5.0
MPEG1	226818_at	767.9	156.3	4.9
MPEG1	226841_at	394.5	81.1	4.9
FYB	205285_s_at	144.7	29.9	4.8
ITGAL	1554240_a_at	152.2	31.9	4.8
IGSF6	206420_at	216.2	45.4	4.8
HLA-DRB1 /// HLA-I	209312_x_at	6458.7	1366.3	4.7
LY9	210370_s_at	21.9	4.7	4.7
STAT1	200887_s_at	3630.1	784.0	4.6
HLA-DMA	217478_s_at	1423.4	312.0	4.6
NLRC5	226474_at	289.8	64.1	4.5
HLA-DRB6	217362_x_at	511.6	113.6	4.5
SIGLEC1	219519_s_at	137.8	30.8	4.5
CYTIP	209606_at	96.5	21.9	4.4
HLA-DRB1 /// HLA-I	204670_x_at	5147.3	1174.1	4.4
AIF1	213095_x_at	258.0	58.9	4.4
BTN3A3	204821_at	895.4	205.2	4.4
BTN3A3	38241_at	756.8	182.4	4.2
LST1	215633_x_at	77.5	19.1	4.1
NCF4	207677_s_at	53.9	13.5	4.0
AIF1	215051_x_at	329.7	83.2	4.0
XAF1	228617_at	369.9	93.9	3.9
IL10RA	204912_at	265.9	68.1	3.9
HLA-E	200904_at	4159.3	1080.4	3.8
BATF2	228439_at	225.5	58.8	3.8
TRIM22	213293_s_at	950.0	252.5	3.8

CARD16 /// CASP1	1552703_s_at	248.9	66.2	3.8
PSMB10	202659_at	647.6	173.9	3.7
TAPBPL	218747_s_at	283.6	76.7	3.7
RASSF5	223322_at	174.5	47.7	3.7
LST1	214181_x_at	103.2	28.3	3.6
FPR3	230422_at	165.9	46.7	3.6
HLA-DMB	203932_at	646.5	183.3	3.5
LILRB2	207697_x_at	102.3	29.1	3.5
IFIT3	229450_at	847.5	242.6	3.5
HLA-E	200905_x_at	7782.5	2237.1	3.5
CD86	210895_s_at	161.7	46.9	3.4
CD48	204118_at	116.7	34.6	3.4
XAF1	206133_at	153.8	45.9	3.4
HLA-DOA	206313_at	45.3	13.6	3.3
HCLS1	202957_at	365.9	109.9	3.3
CARD16	1552701_a_at	224.3	67.4	3.3
SECTM1	213716_s_at	191.9	58.1	3.3
TAPBPL	218746_at	257.8	79.0	3.3
TNFRSF1B	203508_at	166.2	51.3	3.2
AMICA1	228094_at	54.7	17.1	3.2
HLA-J	217436_x_at	3100.4	986.8	3.1
ITGAL	213475_s_at	90.3	29.4	3.1
PSME2	201762_s_at	2069.5	681.6	3.0
PRF1	1553681_a_at	155.0	51.3	3.0
CD70	206508_at	80.5	27.2	3.0
HLA-E	217456_x_at	2502.3	863.9	2.9
TMSB4X	216438_s_at	5518.4	1915.0	2.9
IL15RA	207375_s_at	70.4	25.2	2.8
TRIM69	1568592_at	610.6	218.5	2.8
TAPBP	208829_at	1997.4	723.2	2.8
LILRB1	211336_x_at	86.2	31.4	2.7
HLA-B	211911_x_at	10442.8	3816.9	2.7
LAIR1	208071_s_at	60.1	22.8	2.6
CAPN10	219333_s_at	38.4	14.6	2.6
PTPN6	206687_s_at	159.8	61.1	2.6
IRF9	203882_at	798.8	312.4	2.6
CASP1	206011_at	87.3	34.7	2.5
LGMN	201212_at	1406.5	561.7	2.5
CLEC4A	219947_at	39.8	16.2	2.5
HLA-B	208729_x_at	7820.4	3272.1	2.4
CD68	203507_at	123.7	51.9	2.4
C2	203052_at	1113.0	473.4	2.4
CD14	201743_at	1008.3	431.3	2.3
DOCK8	225502_at	253.0	109.3	2.3
HLA-G	211528_x_at	6898.4	3124.2	2.2
OAS3	218400_at	258.5	129.1	2.0
NCF4	205147_x_at	71.9	36.6	2.0
SP110	209761_s_at	388.0	225.8	1.7
ABHD16A /// LY6G6	224756_s_at	466.5	271.7	1.7
HLA-C	216526_x_at	9153.9	5455.3	1.7
NLK	222590_s_at	118.8	72.1	1.6
B2M	216231_s_at	12216.2	7478.4	1.6
HLA-C	208812_x_at	9160.0	5746.7	1.6
SNX1	214531_s_at	358.3	227.6	1.6
EHD1	209039_x_at	203.1	132.4	1.5
PSMC2	201067_at	340.9	242.1	1.4
B2M	201891_s_at	10258.1	7539.3	1.4

Probe sets underexpressed in immune gene-high tumors

From immune gene analysis*				
Gene symbol	Probeset ID	Average expression		Fold change
		Immune gene-high	Immune gene-low	
ANTXR1	220093_at	2.1	14.3	0.1
RBM15	232971_at	1.5	9.9	0.2
CFHR3	1570228_at	2.1	12.0	0.2
OCLN	209925_at	3.0	16.5	0.2
OPRM1	207994_s_at	1.2	5.9	0.2
PDGFD	219304_s_at	41.2	199.7	0.2
SLC5A8	237503_at	2.0	7.1	0.3
SCARA5	1554705_at	1.4	4.8	0.3
CD44	217523_at	134.9	269.5	0.5
SOS2	217644_s_at	20.2	38.1	0.5
NRARP	226499_at	53.1	96.7	0.5

* The p-values of all genes was highly significant

Table S3. *Pathways associated with overexpressed and underexpressed genes*

Overexpressed genes			
**Pathway**	**Gene count**	**Genes ratio**	**p-value**
Immune System	48	4.3	1.10E-22
Antigen processing and presentation	16	22.5	3.24E-17
Adaptive Immune System	31	5.1	1.60E-15
Allograft rejection	12	34.3	2.22E-15
Graft-versus-host disease	12	32.4	3.88E-15
Type I diabetes mellitus	12	29.3	1.36E-14
Autoimmune thyroid disease	12	24.0	1.69E-13
Viral myocarditis	12	21.4	6.55E-13
Staphylococcus aureus infection	11	20.8	1.12E-11
Interferon Signaling	15	9.5	6.24E-11
Interferon gamma signaling	11	17.5	6.93E-11
Cytokine Signaling in Immune system	18	6.7	9.20E-11
Cell adhesion molecules (CAMs)	14	9.7	2.02E-10
Natural killer cell mediated cytotoxicity	13	10.0	7.72E-10
Antigen processing-Cross presentation	11	13.8	7.73E-10
ER-Phagosome pathway	10	15.9	1.35E-09
Immunoregulatory interactions between a lymphoid and a non-lymphoid cell	10	14.1	4.34E-09
Phagosome	13	8.6	4.35E-09
Antigen Presentation: Folding, assembly and peptide loading of class I MHC	7	29.2	9.31E-09
Interferon alpha/beta signaling	9	15.3	1.46E-08
Underexpressed genes			
**Pathway**	**Gene count**	**Genes ratio**	**p-value**
Prostate cancer	2	2%	3.49E-03
Hepatitis C	2	1%	4.02E-03
Integrin signaling pathway	2	2%	4.46E-03
Regulation of actin cytoskeleton	2	1%	6.55E-03
Gap junction	2	2%	6.68E-03
Focal adhesion	2	1%	7.08E-03
EGFR1	2	0%	2.65E-02

Table S4. *Gene ontology terms associated with overexpressed and underexpressed genes*

GO term	GO ID	Gene count	Gene ratio	p-value
Overexpressed genes				
**Biological process**				
Immune response	GO:0006955	32	8.7	9.53E-26
Innate immune response	GO:0045087	45	3.3	2.99E-20
APP† of peptide antigen via MHC class I	GO:0002474	14	14.1	2.15E-13
Interferon-gamma-mediated signaling pathway	GO:0060333	12	19.7	3.19E-13
Antigen processing and presentation	GO:0019882	12	18.8	4.73E-13
Cytokine-mediated signaling pathway	GO:0019221	18	7.2	1.09E-12
APP† of exogenous peptide antigen via MHC class I, TAP-dependent	GO:0002479	11	14.7	8.92E-11
APP† of exogenous peptide antigen via MHC class I	GO:0042590	11	13.9	1.41E-10
Defense response to virus	GO:0051607	13	8.9	2.96E-10
Type I interferon signaling pathway	GO:0060337	10	15.9	3.19E-10
**Cellular component**				
MHC class I protein complex	GO:0042612	6	50.0	2.62E-09
Integral component of lumenal side of endoplasmic reticulum membrane	GO:0071556	7	29.2	4.81E-09
External side of plasma membrane	GO:0009897	13	6.8	6.98E-09
MHC class II protein complex	GO:0042613	6	37.5	1.57E-08
ER to Golgi transport vesicle membrane	GO:0012507	7	21.9	3.25E-08
**Molecular function**				
Peptide antigen binding	GO:0042605	8	30.8	1.73E-10
Protein binding	GO:0005515	90	0.9	5.32E-09
MHC class I protein binding	GO:0042288	5	41.7	2.16E-07
MHC class II protein complex binding	GO:0023026	5	31.3	1.07E-06
CCR5 chemokine receptor binding	GO:0031730	3	50.0	8.51E-05
Underexpressed genes				
**GO term**	**GO ID**	**Gene Coun**	**Gene ratio**	**p-value**
**Biological process**				
Negative regulation of Wnt protein secretion	GO:0061358	1	100%	1.46E-02
Positive regulation of ERK1 and ERK2 cascade	GO:0070374	2	2%	1.63E-02
Cellular response to morphine	GO:0071315	1	50%	1.75E-02
Positive regulation of monocyte aggregation	GO:1900625	1	50%	1.75E-02
Wound healing involved in inflammatory response	GO:0002246	1	50%	1.75E-02
Blood vessel endothelial cell proliferation involved in sprouting angiogenesis	GO:0002043	1	25%	2.19E-02
Iron ion transmembrane transport	GO:0034755	1	25%	2.19E-02
Positive regulation of heterotypic cell-cell adhesion	GO:0034116	1	25%	2.19E-02
Positive regulation of transcription of Notch receptor target	GO:0007221	1	25%	2.19E-02
Monocyte aggregation	GO:0070487	1	33%	2.19E-02
**Cellular component**				
Apicolateral plasma membrane	GO:0016327	1	9%	2.41E-02
Membrane	GO:0016020	5	0%	2.55E-02
Apical plasma membrane	GO:0016324	2	1%	2.59E-02
Lamellipodium membrane	GO:0031258	1	7%	2.61E-02
Filopodium membrane	GO:0031527	1	8%	2.64E-02
**Molecular function**				
Beta-endorphin receptor activity	GO:0004979	1	100%	1.46E-02
Ferritin receptor activity	GO:0070287	1	100%	1.46E-02
Morphine receptor activity	GO:0038047	1	100%	1.46E-02
Thiopurine S-methyltransferase activity	GO:0008119	1	50%	1.75E-02
Hyaluronoglucosaminidase activity	GO:0004415	1	14%	2.27E-02

†APP: Antigen processing and presentation

Table S5. *List of cancer germline genes, melanocyte differentiation genes, genes associated with pigmentation, melanosome and eye pigmentation*

Cancer-testis genes[a]	Melanocyte differentiation genes[b]	Pigmentation[c]	Melanosome[c]
ACRBP	DCT	ADAMTS20	AHCY
ACTL8	DCT	AP1M1	ANXA11
ADAM29	DCT	AP3D1	ANXA2P2
AKAP3	MLANA	ARL6	ANXA6
AKAP4	MLANA	ASIP	ATP1A1
ARX	TYR	BBS4	ATP6V1B2
ATAD2	TYRP1	BBS5	ATP6V1G2
BAGE	PMEL/GP100	BBS7	BSG
BAGE2 /// BAGE3 /// BAGE5 /// KMT2C	GPR143/OA1	BCL2	CALU
BAGE2 /// BAGE4	RAB38/NY-MEL-1	BCL2L11	CANX
C15orf60		BLOC1S2	CCT4
CASC5		CNO	CTSD
CCDC62		EDN3	ERP29
CCDC83		EDNRB	FASN
CEP290		EN1	FLOT1
CEP55		FIG4	GANAB
COX6B2		FOXD1	GCHFR
CRISP2		FOXD3	GNA13
CSAG2 /// CSAG3		FOXD4	GPNMB
CT45A1 /// CT45A2 /// CT45A3 /// CT45A4 /// CT45A5 /// CT45A6 ///		FOXD4L1	GPR143
LOC101060208 /// LOC101060210 /// LOC101060211		FOXD4L6	HSP90B1
CTAG2		GNA11	HSPA5
CTAGE1		GPR143	ITGB1
CTAGE5 /// MIA2		HPS5	MLPH
CXorf48		HPS6	MMP14
CXorf61		IHH	MYH11
cyclin A1		KITLG	NAP1L1
DDX53		LYST	NCSTN
DKKL1		MAP2K1	OCA2
DMRT1		MITF	PDCD6IP
DPPA2		MKKS	PDIA3
DSCR8		MLPH	PDIA4
ELOVL4		MYO7A	PMEL
FAM133A		NF1	PPIB
FAM46D		OCA2	PRDX1
FATE1		POMC	RAB35
FTHL17		RECQL4	RAB5A
GAGE1 /// GAGE12B /// GAGE12C /// GAGE12D /// GAGE12E ///		SHROOM2	RAB5B
GAGE12F /// GAGE12G /// GAGE12H /// GAGE12I /// GAGE12J ///		SOD2	RAC1
GAGE2A /// GAGE2B /// GAGE2C /// GAGE2D /// GAGE2E /// [illegible]		SOX10	RAN
GAGE1 /// GAGE12C /// GAGE12D /// GAGE12E /// GAGE12F ///		SZT2	RPN1
GAGE12G /// GAGE12H /// GAGE12I /// GAGE12J /// GAGE2A ///		TH	SDCBP
GAGE2B /// GAGE2C /// GAGE2D /// GAGE2E /// GAGE3 /// GAGE4 [illegible]		TYR	SEC22B
[illegible] GAGE12F /// GAGE12G /// GAGE12I /// GAGE4 /// GAGE5 /// GAGE6		TYRP1	SERPINF1
/// GAGE7		VPS33A	SLC1A4
GAGE3		ZIC2	SLC1A5
GPAT2			SLC24A5
GPATCH2			SLC2A1
HEMGN			SLC3A2
HORMAD1			SLC45A2
HORMAD2			SND1
HSPB9			STOM
IGF2BP3			SYNGR1
IGSF11			SYPL1
IL13RA2			SYTL2
KDM5B			TH
KIAA0100			TMED10
KIF20B			TMEM33

KIF2C			TPP1
LDHC			TRPV2
LIPI			TYR
LOC100996400 /// NOL4			TYRP1
LOC101060193 /// XAGE2 /// XAGE2B			YWHAB
LOC101060230 /// MAGEA12			YWHAE
LOC101060424 /// LOC653786 /// OTOA			YWHAZ
LOC253842 /// NR6A1	Eye pigmentation[c]		
LOC440934			
LUZP4	AP3D1		
LYPD6B	GPR143		
LY6K	IHH		
MAEL	OCA2		
MAGEA1	TYR		
MAGEA10			
MAGEA10-MAGEA5 /// MAGEA5			
MAGEA11			
MAGEA8			
MAGEB1			
MAGEB2			
MAGEB4			
MAGEB6			
MORC1			
MSANTD3-TMEFF1 /// TMEFF1			
NXF2 /// NXF2B /// TCP11X1 /// TCP11X2			
ODF2			
ODF3			
PAGE1			
PAGE4			
PAGE5			
PASD1			
PBK			
PIWIL2			
PLAC1			
POTEG /// POTEM			
PRAME			
PRM2			
PRSS50			
PRSS54			
PRSS55			
RBM46			
RGS22			
RHOXF2			
RNF17			
RNF19			
RNF20			
RQCD1			
SAGE1			
SEMG1			
SLCO6A1			
SPA17			
SPACA3			
SPAG1			
SPAG17			
SPAG4			
SPAG8			
SPAG9			
SPANXA1 /// SPANXA2			
SPANXB1 /// SPANXB2 /// SPANXF1			
SPANXC /// SPANXE			
SPATA19			
SPEF2			
SPO11			

SSX1
SSX4 /// SSX4B
SSX5
TAF7L
TDRD1
TEKT5
TEX14
TFDP3
THEG
TMEFF2
TMEM108
TMPRSS12
TPPP2
TPTE
TSGA10
TTK
TULP2
VENTXP1
XAGE1A /// XAGE1B /// XAGE1C /// XAGE1D /// XAGE1E
XAGE3
XAGE4
ZNF165
ZNF645

[a]Source: http://www.cta.lncc.br

[b]Source: http://www.cancerimmunity.org/peptide/

[c]Source: http://amigo1.geneontology.org

Table S6. *Cox univariate analyses for the duration of DFS in uveal melanomas according to clinical, histological, genetic and immune infiltrate parameters*

**Variable**			**n**	**Events**	**HR†**	**95% CI for HR†**	**p-value**
Clinical features	Age	≤ 60 years<br>> 60 years	31<br>55	12<br>26	1.0<br>1.2	0.6-2.4	0.6
	Gender	female<br>male	41<br>45	15<br>23	1.0<br>1.6	0.9-3.1	0.1
	Diameter	≤ 15mm<br>> 15mm	16<br>58	7<br>25	1.0<br>1.0	0.4-2.4	1.0
	Thickness	≤ 8mm<br>> 8mm	15<br>66	8<br>28	1.0<br>0.9	0.4-1.9	0.7
	Location	choroid<br>choroid+CB‡	36<br>50	14<br>24	1.0<br>1.4	0.7-2.7	0.3
	Stage	II<br>III & IV	25<br>50	7<br>26	1.0<br>2.5	1.1-5.9	0.03*
	Extrascleral extension	absent<br>present	79<br>7	32<br>6	1.0<br>3.5	1.5-8.6	0.005*
Histological features	Epithelioid component	≤ 25%<br>> 25%	49<br>36	15<br>23	1.0<br>3.0	1.6-5.8	0.001*
	Mitoses	≤ 10/40hpf<br>> 10/40hpf	41<br>44	10<br>28	1.0<br>4.4	2.1-9.0	< 0.001*
	Necrosis	absent<br>present	75<br>11	29<br>9	1.0<br>3.4	1.6-7.3	0.001*
	Pigmentation	≤ 25%<br>> 25%	66<br>20	28<br>10	1.0<br>1.3	0.6-2.6	0.5
Copy number alterations	M3	absent<br>present	34<br>47	5<br>32	1.0<br>7.0	2.7-17.9	< 0.001*
	i8q	absent<br>present	65<br>16	23<br>14	1.0<br>5.4	2.7-10.8	< 0.001*
	8q+	absent<br>present	41<br>40	9<br>28	1.0<br>5.2	2.4-11.1	< 0.001*
	8p-	absent<br>present	59<br>22	22<br>15	1.0<br>2.6	1.3-5.0	0.005*
	6p+	absent<br>present	55<br>26	28<br>9	1.0<br>0.5	0.3-1.2	0.1
	1p-	absent<br>present	63<br>18	27<br>10	1.0<br>1.2	0.6-2.5	0.6
	LZTS1 deletion	absent<br>present	43<br>38	16<br>21	1.0<br>1.8	1.0-3.5	0.07
	NBL1 deletion	absent<br>present	44<br>37	18<br>19	1.0<br>1.2	0.6-2.2	0.6
Immune	Immune infiltrate	Immune infiltrate-low<br>Immune infiltrate-high	67<br>19	22<br>16	1.0<br>4.6	2.4-9.0	< 0.001*

* p ≤ 0.05 considered significant
‡CB: ciliary body †HR: hazard ratio

Table S7. *Cox univariate and multivariate analyses for the duration of DFS on MLPA probes*

Chromosome/ alteration	Gene	HR	95.0% CI for HR	p-value
Univariate				
1p-	*MFN2*	1.0	0.5-1.9	0.9
	NBL1	1.2	0.6-2.2	0.6
	PTAFR	1.0	0.5-2.0	1.0
	GJB3	0.7	0.4-1.4	0.3
	MUTYH	0.8	0.4-1.6	0.5
	RPE65	1.1	0.6-2.2	0.8
	NOTCH2	1.0	0.5-2.0	0.9
3p-	*CHL1*	2.2	1.1-4.6	0.03*
	VHL	2.7	0.8-8.8	0.1
	C3ORF10	5.6	2.0-15.8	0.001*
	PPARG	2.7	1.4-5.3	0.004*
	XPC	3.1	1.0-10.2	0.06
	MIR128	4.7	2.0-11.4	0.001*
	MLH1	3.7	1.5-9.0	0.003*
	CTNNB1	2.1	1.0-4.3	0.04*
	RBM5	2.7	1.3-5.7	0.01*
	BAP1	3.3	1.2-9.3	0.03*
	FHIT	7.1	2.2-23.3	0.001*
	ROBO1	2.3	0.8-6.5	0.1
3q-	*PROS1*	2.6	1.2-5.8	0.02*
	CASR	2.6	1.2-5.6	0.01*
	MME	6.1	2.1-17.2	0.001*
	OPA1	2.6	1.2-5.6	0.01*
6p+	*ECI2*	0.7	0.3-1.3	0.2
	DCDC2	0.7	0.4-1.3	0.3
	CDKN1A	0.5	0.3-1.0	0.06
	RUNX2	0.6	0.3-1.2	0.2
6q-	*CTGF*	0.8	0.4-1.7	0.6
	IGF2R	0.8	0.4-1.5	0.4
8p-	*LZTS1*	1.8	1.0-3.5	0.07
	NRG1	2.8	1.5-5.5	0.002*
8q+	*RP1*	5.3	2.5-11.0	< 0.001*
	MYC	6.6	0.9-48.1	0.06
	ASAP1	6.1	1.9-19.9	0.003*
Multivariate - final model				
NBL1 deletion		5.3	2.2-13.0	< 0.001*
LZTS1 deletion		5.3	2.2-12.8	< 0.001*
M3		11.0	3.4-35.7	< 0.001*
8q+		3.9	1.6-9.2	0.002*

* p ≤ 0.05 considered significant

Table S8. *Cox multivariate analyses for the duration of DFS in uveal melanomas for the various models constructed*

Before backward stepwise				***Final model***			
**Variables in the model at the beginning**	**HR†**	**95.0% CI for HR**	**p-value**	**Variables retained in the final model**	**HR†**	**95.0% CI for HR**	**p-value**
**Classic**				**Classic**			
Immune infiltrate	0.9	0.3-2.8	0.89	Mitoses	3.5	1.4-8.3	0.005*
Gender	0.7	0.3-1.7	0.40	Extrascleral extension	8	2.5-25.2	<0.001*
Stage	0.9	0.3-2.6	0.92	M3	3.1	1.1-9.1	0.04*
Epithelioid component	1.6	0.6-4.1	0.32	Isochromosome 8q	4.2	1.7-10.3	0.002*
Mitoses	2.5	0.9-7.1	0.09				
Necrosis	2.7	0.8-8.7	0.10				
Extrascleral extension	6.6	1.2-36.9	0.03				
M3	5.1	1.0-25.4	0.05				
8q+	1.2	0.4-4.0	0.73				
Isochromosome 8q	3.1	0.1-77.0	0.50				
8p-	1.1	0.1-13.1	0.95				
LZTS1deletion	1.4	0.3-6.4	0.63				
6p+	1.4	0.5-4.3	0.53				
**Clinical**				**Clinical**			
Age	1.0	0.5-2.1	0.9	Stage	2.5	1.1-5.8	0.03*
Gender	1.6	0.8-3.3	0.2	Extrascleral extension	3.2	1.2-8.3	0.02*
Stage	3.9	1.2-12.3	0.02*				
Location	0.6	0.2-1.6	0.3				
Extrascleral extension	2.0	0.6-6.5	0.3				
**Histology**				**Histology**			
Epithelioid component	2.0	1.0-4.0	0.05*	Epithelioid component	2.1	1.0-4.1	0.04*
Mitotic activity	3.0	1.4-6.6	0.006*	Mitotic activity	2.9	1.3-6.5	0.008*
Pigmentation	1.3	0.6-2.7	0.5	Necrosis	2.1	1.0-4.6	0.06
Necrosis	2.1	0.9-4.5	0.07				
**CNA**				**CNA**			
8p-	1.5	0.5-4.6	0.5	M3	11.0	3.4-35.7	< 0.001*
M3	14.5	3.7-55.9	< 0.001*	8q+	3.9	1.6-9.2	0.002*
8q+	4.4	1.7-11.2	0.002*	*LZTS1* deletion	5.3	2.2-12.8	< 0.001*
6p+	1.5	0.5-4.3	0.5	*NBL1* deletion	5.3	2.2-13.0	< 0.001*
LZTS1 deletion	4.3	1.4-13.6	0.01*				
1p-	1.5	0.6-4.0	0.4				
NBL1 deletion	5.1	1.8-14.5	0.002*				
**Immune-CNA**				**Immune-CNA**			
8p-	1.5	0.5-4.6	0.5	M3	9.2	2.7-31.1	< 0.001*
M3	14.5	3.7-55.9	< 0.001*	8q+	3.6	1.5-8.7	0.005*
8q+	4.4	1.7-11.2	0.002*	*LZTS1* deletion	4.6	1.8-11.6	0.001*
6p+	1.5	0.5-4.3	0.5	*NBL1* deletion	5.8	2.3-14.8	< 0.001*
LZTS1 deletion	4.3	1.4-13.6	0.01*	Immune infiltrate ‡	1.6	0.7-3.7	0.2
1p-	1.5	0.6-4.0	0.4				
NBL1 deletion	5.1	1.8-14.5	0.002*				
Immune infiltrate							

* $p \le 0.05$ considered significant, ‡ Immune forced into the model

†HR: hazard ratio

Table S9. *Predictive accuracies of Cox univariate and multivariate models*

Variable	Cox PHA†	AUC at fixed time points (months)					Integrated AUC (C^T) (months)					Harrell's C-index	SE§
		12	24	36	48	64	12	24	36	48	64		
**Univariate**													
Immune infiltrate	0.77	0.66	0.65	0.628	0.551	0.502	0.68	0.67	0.66	0.66	0.65	0.653	0.03
Age‡	0.03	0.54	0.54	0.583	0.607	0.7	0.56	0.55	0.55	0.56	0.57	0.502	0.04
Gender	0.34	0.56	0.56	0.572	0.561	0.539	0.56	0.56	0.56	0.56	0.56	0.578	0.04
Diameter	0.89	0.5	0.49	0.513	0.502	0.516	0.5	0.5	0.5	0.5	0.5	0.503	0.04
Thickness	0.22	0.5	0.5	0.518	0.511	0.515	0.5	0.5	0.51	0.51	0.51	0.501	0.04
Location	0.71	0.54	0.54	0.553	0.542	0.544	0.54	0.54	0.54	0.54	0.54	0.546	0.04
Stage	0.18	0.59	0.59	0.62	0.609	0.609	0.59	0.59	0.59	0.59	0.59	0.610	0.04
Extrascleral extension	0.80	0.56	0.54	0.533	0.5	0.502	0.57	0.57	0.56	0.56	0.55	0.559	0.02
Epithelioid	0.32	0.64	0.64	0.628	0.631	0.619	0.64	0.64	0.64	0.64	0.64	0.651	0.04
Mitotic activity‡	0.03	0.72	0.63	0.56	0.572	0.584	0.73	0.72	0.69	0.68	0.67	0.690	0.04
Necrosis	0.97	0.59	0.57	0.569	0.535	0.551	0.59	0.58	0.58	0.58	0.58	0.579	0.03
Pigmentation	0.33	0.53	0.51	0.53	0.515	0.511	0.52	0.52	0.52	0.52	0.52	0.519	0.04
M3	0.83	0.68	0.69	0.725	0.715	0.721	0.67	0.68	0.69	0.69	0.69	0.692	0.04
i8q	0.78	0.65	0.64	0.585	0.561	0.502	0.68	0.67	0.66	0.65	0.64	0.654	0.03
8q+	0.42	0.68	0.69	0.711	0.695	0.707	0.68	0.69	0.69	0.69	0.69	0.699	0.04
8p-	0.29	0.59	0.6	0.587	0.586	0.583	0.61	0.61	0.6	0.6	0.6	0.617	0.04
6p+	0.34	0.56	0.56	0.577	0.568	0.549	0.56	0.56	0.56	0.56	0.56	0.576	0.04
1p-	0.10	0.52	0.51	0.529	0.518	0.555	0.51	0.51	0.51	0.51	0.51	0.499	0.04
LZTS1 deletion	0.16	0.57	0.57	0.576	0.577	0.585	0.58	0.58	0.58	0.58	0.58	0.594	0.04
NBL1 deletion	0.09	0.52	0.51	0.53	0.52	0.542	0.51	0.52	0.52	0.52	0.52	0.502	0.04
**Multivariate models**													
Clinical	0.43	0.63	0.62	0.618	0.608	0.608	0.64	0.64	0.63	0.63	0.63	0.631	0.05
Histology	0.20	0.73	0.73	0.722	0.699	0.715	0.73	0.73	0.73	0.73	0.72	0.743	0.05
CNA	0.22	0.8	0.82	0.832	0.788	0.801	0.82	0.82	0.83	0.82	0.82	0.797	0.05
Immune-CNA	0.33	0.82	0.83	0.837	0.794	0.797	0.83	0.83	0.83	0.83	0.83	0.809	0.05
Classic	0.50	0.82	0.83	0.801	0.798	0.758	0.84	0.83	0.83	0.83	0.82	0.844	0.05
**Cassoux model¶**													
M3/8q+	0.65	0.74	0.75	0.778	0.759	0.743	0.73	0.74	0.75	0.75	0.75	0.748	0.05

‡Local Cox

†PHA: proportional hazards assumption; §SE: standard error

¶From Cassoux,et al. Br J Ophthalmol. 2013 Oct 29
