# Supplementary material for: DNA alteration‐based classification of uveal melanoma gives better prognostic stratification than immune infiltration, which has a neutral effect in high‐risk group
Source: Cancer Med. 2019 Apr 25;8(6):3036–46. doi: 10.1002/cam4.2122 (PMC6558590; doi:10.1002/cam4.2122)
Supplement: Supplementary file 3 [file CAM4-8-3036-s003.pdf]

## Supplementary methods

### Tumor series

For 89/91 tumors, clinico-radiological data and follow-up information were available and obtained from hospital and general practitioner records and histopathological details from haematoxylin and eosin (H&E) sections. Age at operation varied from 35 years to 90 years with a mean of 64.6 years and a median of 67 years. The male to female ratio was 1.1. The median basal diameter was 18mm and median thickness was 11.1mm with tumors mainly belonging to T3 and T4 (91%) categories. TNM staging was performed according to 7$^{th}$ edition of American Joint Committee on Cancer guidelines [1]. Retinal detachment status was available for 81 tumors and present in 79 (97.5%) of them. Haematoxylin and eosin sections were assessed for tumor cell morphology, mitotic activity, extrascleral extension and histological typing to classify them according to modified Callender's classification[2]. The epithelioid component constituted up to 25% of the tumoral population in 51 tumors (58%), and was greater in the remaining tumors 37 (42%). Mitotic activity was up to 10/40 high power fields (hpf) in 43 tumors (48.9%) and higher in 45 (51.1%). Necrosis was present in 11 tumors (12.4%) and absent in 78 tumors (87.6%) (**Table S1**).

### RNA Extraction

For the 15 snap-frozen samples, total RNA was extracted using guanidine isothiocyanate/cesium chloride procedure [3]. The first-strand cDNA was synthesized from 2µg of total RNA with M-MLV reverse transcriptase (catalog # 28025) (Invitrogen, Life Technologies) and random hexamer primer (Fermentas), as recommended by manufacturer.

For 21 samples stored in RNA later (-80°C), RNA was extracted using NucleoSpin RNA (catalog # 740955) (Macherey-Nagel, Düren, Germany). For each tumor, 20 cryosections of 20µm thickness were used for extraction. Isolation of total RNA was performed using NucleoSpin RNA (catalog # 740955) (Macherey-Nagel, Düren, Germany) as per the

manufacturer's guidelines. In brief, the cryosections were homogenized with RA1 buffer and beta-mercaptoethanol and filtered using NucleoSpin filters. The filtrate was mixed with 70% ethanol and bound to NucleoSpin RNA column. The column was washed and dried using buffers RAW2 and RA3, followed by elution using RNase-free water. Total RNA was treated with Turbo DNA-free™ kit (catalog # AM1907) (Ambion, Life Technologies, Carlsbad, CA) to remove the residual DNA. RNA was quantified using NanoDrop (ThermoScientific, Wilmington, DE). The $A_{260}/A_{280}$ ratio for the samples ranged from 1.88-1.99. The melanin concentration in RNA was assessed by measuring the absorbance at 320nm and samples with absorbance >0.1 were re-purified on RNA column. The first strand cDNA was synthesized from 1µg of total RNA using RevertAid H minus first strand cDNA synthesis kit and random hexamer primer (catalog # K1632) (Fermentas, ThermoScientific), as per manufacturer's guidelines.

**Array hybridization**

RNA was quantified using NanoDrop (ThermoScientific, Wilmington, DE) and samples with $A_{260}/A_{280}$ ratio between 1.70-1.83 were included. Also, only samples with intact RNA indicated by sharp, clear bands for 18S and 28S rRNA on agarose gels were utilized. Human Genome U133 Plus 2.0 arrays (Affymetrix, Santa Clara, CA) were used to generate gene expression profiles from 5µg of total RNA following manufacturer's protocol. The intensity files were obtained by processing the images using Microarray suite 5.0 gene expression software. Probe sets present in <90% of the samples were excluded from analysis.

**Array data analysis**

Additional normalization was performed to reduce variation between individual hybridizations. Therefore, the mean intensity of each probe set was defined based on the intensity of all tumors. Then, individual intensities were divided by their respective mean

intensity and converted to log2. The first 1500 probe sets with highest standard deviation (SD) were submitted for statistical analysis using TIGR MultiExperiment Viewer platform (MeV4.8.1, http://www.tm4.org/mev/)[4]. Subgroups of uveal melanoma were unraveled by performing hierarchical clustering with Pearson's correlation. Estimation of sampling distribution was assessed by bootstrapping, using 100 iterations. Differentially expressed probe sets were identified by T-test applying high stringency (5000 permutations, p-value 0.001) and low stringency (1000 permutations, p-value 0.01) conditions, with adjusted Bonferroni method of p-value correction[5].

The annotation of statistically significant probe sets was performed using InnateDB[6] by applying a false-discovery rate cut-off of 0.05 and p-value was correction by Benjamini-Hochberg method.

**Immune genes**

The immunologically relevant genes were compiled from three databases: 5998 genes from ImmPort (https://immport.niaid.nih.gov), 1773 genes related to "immune system process" from Gene Ontology database (http://amigo.geneontology.org) and 1326 immunologically important genes from Wiki for Immunology (http://wiki.geneontology.org/index.php/Immunology). There were a total of 6487 genes after accounting for common genes between databases and 4840 genes with available probe sets (n=11,103) were included for further analysis.

**GBP1 immunohistochemistry**

4μm sections were deparaffinized, endogenous peroxidase activity was blocked with 3% hydrogen peroxide and heat-induced epitope retrieval was performed in 0.1M, pH 6.0 citrate buffer at 100°C for 75 minutes. Following blocking of aspecific binding with 10% normal goat serum for 30 minutes, endogenous biotins were neutralized with Avidin/Biotin blocking

kit (catalog # SP-2001) (Vector Laboratories, Burlingame, CA) and the sections were incubated overnight with unlabeled GBP1 primary antibody. After washing, they were incubated with secondary antibody, 1:500 biotinylated anti-mouse IgG (catalog # BA-9200) (Vector laboratories), followed by incubation with 1% streptavidin-AP-conjugate (catalog # 11089161001) (Roche Diagnostics) for 30 minutes each. Revelation was performed using Sigma Fast Fast Red TR/Napthol AS–MX (catalog # F4648) (Sigma-Aldrich, St. Louis, MO) in dark for 30 minutes. The slides were counterstained with haematoxylin and coverslipped using an aqueous mounting medium. All reactions were carried out at room temperature.

**GBP1 quantification**

Digital images of GBP1-stained slides were obtained at 20X magnification using a whole slide scanner, LeicaSCN400 (Leica Microsystems GmbH, Wetzlar, Germany). The images were stored and managed using a web-enabled image server, Digital Image Hub (DIH) (SlidePath, Leica). Quantification of GBP1 was performed using the image analysis software, Tissue IA 2.0 (SlidePath, Leica) using 'measure stained area' algorithm. First, the total tumor area (reference area) to be analyzed was outlined manually using the annotation tool. Image segmentation was performed using two thresholds that were manually set. The first was a tissue segmentation threshold to separate the tumor tissue from background. The second was a color definition threshold to define the positively stained pixels. In order to ensure that the latter adequately reflected all positive staining, it was tested on multiple areas within the same slide and on multiple different slides. The output results included: total area of the annotated tumor (reference area), positively stained tumor area, average staining intensity and concentration of the stain within the tissue sample. The ratio of the positive tumor area with respect to the reference tumor area was computed and was referred to as the GBP1 labeling index[7].

**Quantitative real-time reverse transcription polymerase chain reaction (RT-qPCR)**

The PCR mix was prepared as follows: PCR-grade water 3.4µl, forward and reverse primers 0.8µl each (5µM) and master mix 10µl. For one reaction, 15µl of PCR mix and 5µl of cDNA (2ng/µl concentration) template was used. The LightCycler experimental run protocol was: denaturation program (95°C for 10 min), amplification and quantification program repeated 50 times (95°C for 10s, annealing temperature primer-dependent for 15s, 72°C for 15s), melting curve program (65-97°C) and cooling step to 40°C. All samples were tested in triplicate, along with no-DNA controls. The specificity of the amplified PCR product was assessed by performing melt curve analysis for both target and reference genes. Standard curves were generated for each target and reference genes and were used for quantification by taking PCR efficiencies (E) into consideration. Crossing point (Cp) in an amplification reaction is the cycle at which the fluorescence of a sample rises above the background fluorescence. The relative expression of target genes was determined using the E-method of relative quantification. The mean Cp for target and reference genes was obtained for all samples. The relative expression ratio of the target gene for each sample was calculated using the formula: $(E_{target})^{\Delta Cp\ target} / (E_{reference})^{\Delta Cp\ reference}$ [8]. *CSNK2B* was used as the reference gene for normalization.

**Multiplex ligation-dependent probe amplification (MLPA)**

MLPA was performed on FFPE uveal melanomas. FFPE cortisectomies from epilepsy surgeries were used as controls. The H&E slides were reviewed to ensure samples consisted of ≥ 80% tumor cells. The tumor and control tissue were micro-dissected from 10-15, 10µm FFPE sections and stored at 4°C. Following deparaffinization, tissue lysis and protein digestion were performed. For this, the tissue was incubated in 200µl of lysis buffer (10mM TRIS, 1mM EDTA pH 8.5, 0.01% Tween-20) containing 5% Chelex-100 beads (Code # 142-1253, Bio-Rad, Hercules, CA) and 2µg/µl of Proteinase K (Code # P2308, Sigma-Aldrich,

St.Louis, MO), overnight at 56°C with continuous shaking. This was followed by inactivation of proteinase K at 100°C for 10 minutes and centrifugation at 13,000 rpm for 10 minutes[9]. The supernatant containing the DNA was collected and purified with QIAquick PCR purification kit using the manufacturer recommended microcentrifuge protocol (Qiagen, Valencia, CA). The DNA concentration was assessed by NanoDrop 1000 spectrophotometer (Thermo Scientific Wilmington, DE). The SALSA MLPA kit P027-C1 uveal melanoma (MRC Holland, Amsterdam, Netherlands) was used to identify chromosomal abnormalities in 89 tumor samples. The DNA was diluted in TE (10mM Tris-HCL pH 8.2, 0.1mM EDTA). The total DNA concentration was ~200-250 ng in a volume of 5µl. In each MLPA assay, two to three non-tumor controls were incorporated. The reactions were carried out as per the manufacturer’s recommendations in a C1000 thermal cycler (Bio-Rad). A mixture of 0.7µl of PCR product, 9µl of Hi-Di formamide (Applied Biosystems, Carlsbad, CA) and 0.2 µl GeneScan-500 LIZ size standard (Applied Biosystems) was analyzed by capillary electrophoresis on an ABI-3130XL genetic analyzer (Applied Biosystems). Fragment analysis was performed using GeneMarker v2.2 software (Softgenetics, State College, PA). The peak heights and the sizes of various probes were determined. The peak intensities were normalized by population normalization. The reference sample was calculated from the median intensities of non-tumor controls with standard deviation of ≤0.1 for both internal control and sample probes. For each probe, a ratio was obtained by comparing the peak heights of the samples to that of the reference. Loss of DNA was defined as a ratio of ≤0.88 and gain as ≥1.24. This cut-off was calculated based on the average ratio of all probes on chromosome 3 (for loss) and on chromosome 8q (for gain) in tumors displaying mosaicism by CGH. The chromosomal aberrations were considered as absent or present based on the number and location of aberrant probes.

**Statistical methods**

Continuous variables are reported as mean (standard deviation) and median (range), and are also categorized; binary and categorical variables are reported as frequencies and relative frequencies. Cox proportional hazards regression model was used to investigate the association between each variable and disease-free survival (DFS).

DFS was defined as time from enucleation to radiological diagnosis of metastases for patients developing metastases or death. Patients still alive and metastases-free at the time of analysis were included only if at least 24 months post-enucleation follow-up was available.

*i. Variable selection for Cox multivariate analysis*

All categorical covariates were transformed into numeric codes before they were entered into Cox model. Potential prognostic biomarkers were first investigated in univariate analyses. For each category of biomarkers (clinical features, histological features, immune infiltrate, copy number alterations), all potential prognostic factors were then studied in a multivariate Cox proportional hazards model and a final model was constructed based on a stepwise variable selection procedure. A classic multivariate model, including variables from different categories of biomarkers, was constructed via a stepwise variable selection procedure (starting from a model including all variables with p-value of at least 0.1 in the univariate analysis). Model minimization was performed by backward stepwise conditional likelihood ratio (LR) method in SPSS, except for immune-copy number alteration model where the variable, immune score was forced into the model using enter method in SPSS. The default probability for variable entry (0.05) and removal (0.1) with 20 iterations were used.

*ii. Assessment of model performance*

The performance of both univariate and multivariate Cox models were quantified using Harrell’s C-index, a measure of concordance. A C-index of 0.5 indicates that the model has no discriminative ability, whereas a C-index of 1 indicates that the model perfectly

distinguishes between those with poor prognosis (early metastasis) from those with good prognosis (late metastasis). The models were also evaluated in a time-specific manner by constructing time-dependent ROC curves [10] at fixed time-points (12, 24, 36, 48 and 64 months) and AUC plots up to a specified time-point (12, 24, 36, 48 and 64 months) by integrating AUC values to obtain a global concordance ($C^T$). The assumption of proportional hazards (PHA) was tested and retained for all categorical variables except for age and mitotic activity. However, for the latter two variables, after investigation of the KM plots we decided to continue with the assumption of proportional hazards. Meanwhile, the time-dependent ROC curves and AUC plots for age and mitotic activity were constructed using Local Cox method [10]. The R code for computation of ROC curves was downloaded from http://faculty.washington.edu/heagerty/Software/SurvROC/RisksetROC.

*iii. Prognostic indices*

The prognostic score was based on the estimated regression coefficient of all variables in the final Cox model retained. The score of each variable was its estimated coefficient rounded to the first decimal point (using Microsoft Excel Round function) and multiplied by 10. Individual patient score was obtained by adding up the scores of unfavourable factors present. The samples were split into risk-groups based on tertiles (three groups) and quartiles (four groups). The KM plots of risk groups were compared by log-rank test (both global and pair-wise comparisons with Benjamini-Hochberg adjustment of p-value) (**Table M3**).

*iv. Leave-one-out cross-validation*

The misclassification error associated with each of the models was estimated by leave-one-out method. In each iteration, one observation was omitted and the prognostic score of the omitted observation was predicted based on the model built on the (n-1) other observations. This was repeated for all observations, resulting in cross-validated scores. The samples were split into risk groups based on the cross-validated scores using the same cut-off as used for

the original models. The prediction was considered as 'true' if the predicted and original risk-groups were the same and as 'false' if, they were different. The misclassification error rate was determined based on the number of false observations.

*v. Nomogram*

Nomograms were constructed as per[11].

**External validation of CNA and immune-CNA models on TCGA data**

The copy number alterations, tumor-infiltrating-lymphocyte (TIL) density, tumor-associated-macrophage (TAM) density, time to metastasis/last follow-up information were obtained from data supplement of Robertson, et al[12]. The combined TIL and TAM densities were taken into consideration to categorize tumors as immune-high and immune-low groups (**Table M4**). The LZTS1 deletion and NBL1 deletion status was obtained from cBioPortal[13,14].

***Table M1. Details of antibodies used for immunohistochemistry***

| Primary antibody | Dilution | Incubation time | Clone | Product code | Company |
|---|---|---|---|---|---|
| CD3 | 1:250 | 120 minutes | - | A0452 | Dako, Glostrup, Denmark |
| CD4 | 1:50 | 100 minutes | 4B12 | NCL-CD4-368 | Novocastra, Newcastle upon Tyne, UK |
| CD8 | 1:24 | 48 minutes | CD8/144B | M7103 | Dako |
| CD163 | 1:300 | 72 minutes | 10D6 | NCL-CD163 | Novocastra |
| HLA-DRA | 1:50 | 36 minutes | TAL1.B5 | M0746 | Dako |
| GBP1 | 1:300<br>1:900 | overnight | 4D10 | ab109995 | Abcam, Cambridge, UK |

## *Table M2. Primer sequences used for RT-qPCR*

F: forward; R: reverse

| Gene | Orientation | Primer sequence | Product size | Annealing temperature | Company |
|---|---|---|---|---|---|
| CXCL9 | F | 5'-GCACCAACCAAGGGACTATC-3' | 192 bp | 57°C | Integrated DNATechnologies (IDT) Coralville, IA |
| | R | 5'-GCTTTTTCTTTTGGCTGACC-3' | | | |
| GBP1 | F | 5'-TGTTGCAGGAAATGCAAAGA-3' | 179 bp | 57°C | IDT |
| | R | 5'-TCCCTCTTTTAGTAGTTGCTCCTG-3' | | | |
| RARRES3 | F | 5'-TGGACCATGAGTACCAACCA-3' | 181 bp | 57°C | IDT |
| | R | 5'-GCCACACCAACTTCAACCTT-3' | | | |
| STAT1-β-transcript | F | 5′-ATGGGTGGAGCGGTCCCAGAAC-3′† | 369 bp | 60°C | IDT |
| | R | 5′-AGCATCTTCAACAGGCCCCAGCC-3′† | | | |
| PSMB9 | F | 5'-CAGCTGCTGATGCCCAAG-3' | 437 bp | 60°C | IDT |
| | R | 5'-CAGTTCATTGCCCAAGATGA-3' | | | |
| CSNK2B | F | 5'-GGCAATGAATTCTTCTGTGAA-3' | 271 bp | 60°C | Eurogentec Seraing, Belgium |
| | R | 5'-AACCAAAGTCTCCTTGCTGGT-3' | | | |

†From: Ascierto ML, et al, Breast Cancer Res Treat 2012; 131:871-80

## *Table M3. Prognostic score calculation and cut-off estimation for multivariate models*

| Model | Variable | RC† | Score (RC multiplied by 10) | Tertile cut-off | Quartile cut-off |
|---|---|---|---|---|---|
| CNA | Monosomy 3 | 2.4 | present = 24 | < 31<br>31-51<br>> 51 | < 18<br>18-38<br>39-54<br>> 54 |
| | | | absent = 0 | | |
| | 8q gain | 1.4 | present = 14 | | |
| | | | absent = 0 | | |
| | *LZTS1* deletion | 1.7 | present = 17 | | |
| | | | absent = 0 | | |
| | *NBL1* deletion | 1.7 | present = 17 | | |
| | | | absent = 0 | | |
| Immune-CNA | Immune score | 0.5 | present = 5 | < 31<br>31-47<br>> 47 | < 19<br>19-35<br>36-49<br>> 49 |
| | | | absent = 0 | | |
| | Monosomy 3 | 2.2 | present = 22 | | |
| | | | absent = 0 | | |
| | 8q gain | 1.3 | present = 13 | | |
| | | | absent = 0 | | |
| | *LZTS1* deletion | 1.5 | present = 15 | | |
| | | | absent = 0 | | |
| | *NBL1* deletion | 1.8 | present = 18 | | |
| | | | absent = 0 | | |

†RC: Regression co-efficient

***Table M4. Assignment of immune-groups to TCGA tumors***

| *TIL/TAM density* | *Immune-group* |
|---|---|
| Mild/Mild | Immune-low |
| Mild/Moderate | Immune-low |
| Mild/Heavy | Immune-low |
| Moderate/Mild | Immune-low |
| Moderate/Moderate | Immune-high |
| Moderate/Heavy | Immune-high |
| Heavy/Mild | Immune-low |
| Heavy/Moderate | Immune-high |
| Heavy/Heavy | Immune-high |

## References

1. Sobin LH, Gospodarowicz MK, Wittekind C. *TNM classification of malignant tumours.* 7th ed. ed. Oxford: Wiley-Blackwell; 2010.
2. Font R, Croxatto J, Rao N. *Tumors of the eye and ocular adnexa.* Vol Fascicle 5. Washington DC: American Registry of Pathology; 2006.
3. Cipponi A, Mercier M, Seremet T, et al. Neogenesis of lymphoid structures and antibody responses occur in human melanoma metastases. *Cancer Res.* 2012;72(16):3997-4007.
4. Saeed AI, Sharov V, White J, et al. TM4: a free, open-source system for microarray data management and analysis. *Biotechniques.* 2003;34(2):374-378.
5. Palm T F-BD, Chapon F, Lacroix C, Gray F, Scaravilli F, Ellison DW, Salmon I, Vikkula M, Godfraind C. Expression profiling of ependymomas unravels localization and tumor grade-specific tumorigenesis. *Cancer.* 2009;115(17):3955-3968.
6. Breuer K, Foroushani AK, Laird MR, et al. InnateDB: systems biology of innate immunity and beyond--recent updates and continuing curation. *Nucleic Acids Res.* 2013;41(Database issue):D1228-1233.
7. Decaestecker C, Lopez XM, D'Haene N, et al. Requirements for the valid quantification of immunostains on tissue microarray materials using image analysis. *Proteomics.* 2009;9(19):4478-4494.
8. Uebelhoer M, Natynki M, Kangas J, et al. Venous malformation-causative TIE2 mutations mediate an AKT-dependent decrease in PDGFB. *Hum Mol Genet.* 2013;22(17):3438-3448.
9. de Jong AE, van Puijenbroek M, Hendriks Y, et al. Microsatellite instability, immunohistochemistry, and additional PMS2 staining in suspected hereditary nonpolyposis colorectal cancer. *Clin Cancer Res.* 2004;10(3):972-980.
10. Heagerty PJ, Zheng Y. Survival model predictive accuracy and ROC curves. *Biometrics.* 2005;61(1):92-105.
11. Harrell FE. *Regression modeling strategies : with applications to linear models, logistic regression, and survival analysis.* New York: Springer; 2001.
12. Robertson AG, Shih J, Yau C, et al. Integrative Analysis Identifies Four Molecular and Clinical Subsets in Uveal Melanoma. *Cancer cell.* 2017;32(2):204-220.e215.

13. Cerami E, Gao J, Dogrusoz U, et al. The cBio cancer genomics portal: an open platform for exploring multidimensional cancer genomics data. *Cancer Discov.* 2012;2(5):401-404.
14. Gao J, Aksoy BA, Dogrusoz U, et al. Integrative analysis of complex cancer genomics and clinical profiles using the cBioPortal. *Sci Signal.* 2013;6(269):pl1.
